# Supporting crop yields under climate change by engineering innate soil microbiomes

**DOI:** 10.1128/aem.00437-26

**Published:** 2026-07-13

**Authors:** Anaïs Chanson, Iain J. Gould, Åsgeir R. Almås, Ana Marta Paz, Nádia L. Castanheira, João F. Antunes, Andy Barker, Matthew R. Goddard

**Affiliations:** 1 School of Natural Sciences, University of Lincoln, Lincoln, United Kingdom; 2 Lincoln Institute for Agri-Food Technology, University of Lincoln, Lincoln, United Kingdom; 3 Faculty of Environmental Sciences and Natural Resource Management, Norwegian University of Life Science, Ås, Norway; 4 Instituto Nacional de Investigação Agrária e Veterinária (INIAV), Soil Lab, Oeiras, Portugal; 5 GREEN-IT Bioresources for Sustainability, ITQB NOVA 98819 , Oeiras, Portugal; 6 Barworth Research Ltd, Heckington, United Kingdom; University of Minnesota Twin Cities, St. Paul, Minnesota, USA

**Keywords:** EJP soil, microbiome rescue, agriculture, microbiome engineering, yield protection, soil salinity, irrigation, climate change, bacteria, fungi, DNA, RNA

## Abstract

We test the idea that innate agricultural soil microbiomes can be engineered to help support crops under climate change conditions. Salinization is one of the key drivers of agricultural soil degradation globally, and rising sea levels combined with decreasing rainfall are exacerbating this threat to food production. Changes in soil microbial community structures measured from DNA and functions and fitness measured from RNA and labeled amino acid uptakes support the hypothesis that the deliberate use of part-saline irrigation adapts soil microbiomes to increased salinities. While saline irrigation suppressed crop establishment in some cases, this microbiome response combined with inferences of increased microbial nutrient cycling and energy management correlates with final crop yield data and supports the hypothesis that engineered soil communities have helped protect yields under increased salinity conditions. This work provides evidence of the efficacy of a novel pragmatic, cost-effective innate soil microbiome engineering intervention for optimizing and securing food systems for future climate change conditions.

**IMPORTANCE:**

The consequences of climate change comprise significant and increasing threats to sustainable global food security. The salinization of agricultural soils is one of the main threats to agricultural production globally: this currently affects around 30% and is predicted to affect 50% of agricultural land by 2050. We show innate agricultural soil microbial communities can be engineered by low levels of saline irrigation. The engineered soil communities are better able to tolerate saline conditions and are inferred to have better nutrient turnover, and this correlates with protecting crop yields of established plants under saline conditions. This shows that it is possible for growers and farmers to “teach” or “prepare” soil microbiomes to become more tolerant of elevated soil and irrigation salinities predicted due to climate change in a way that will also meet sustainable food security requirements.

## INTRODUCTION

Salinization currently affects around 30% of agricultural soils and is one of the main threats to agricultural production globally (1, 2): this threat is rising as sea levels do (3) and is predicted to affect 50% of agricultural soils by 2050 (4). Rising sea levels are also driving saline water into freshwater aquifers and drainage ditches used for agricultural irrigation, which is compounding the soil salinization problem (5, 6) as the frequency, intensity, and duration of droughts are also increasing (2, 7–9). This consequence of climate change, therefore, comprises a significant and increasing threat to food security globally.

Salinity refers to the total concentration of all soluble salts (dissolved inorganic ions) and is often determined by electrical conductivity in dS/m, with soils > 2 dS/m considered saline (10), and FAO guidelines (11) suggest irrigation water has no more than 3 dS/m. Salinity affects both soil and crop components of agricultural systems. Elevated sodium ion concentrations damage soil structure (1, 12–17) and increase soil particle dispersion, which alters soil organic matter availability (18). Higher levels of sodium ions not only impose osmotic stresses for crops, which reduces water uptake, but also reduce the uptake of nutrients essential for growth; excess sodium ions can also reduce seed germination (4, 10). In general, existing work shows elevated soil salinities reduce yields in a range of crops, but these studies have mostly been conducted in arid climates (1, 12–17).

Bacteria and fungi are the main drivers of soil nutrient cycles that underpin soil fertility and crop yields (19), but the effects of salinity on soil microbiomes are not well characterized. Older work using gross respiration and growth indicators reported that high salinity exerts a strong effect on microbes after 1-h incubations (20–22). There are only a few studies evaluating the effects of salinity on soil microbial community structures. One UK study showed that less than 2 weeks of flooding with seawater changed microbial community structure and enzyme activities across a saltmarsh to pasture gradient (23). A mesocosm study in the United States showed that the rhizosphere microbiome structure was significantly affected in soils adjusted to 4 dS/m, with bacteria more affected than fungi (24). Other work reports inconsistent responses for major phyla: Actinobacteria and Chloroflexi abundances increased with salinity in arid soils on the edge of the Tengger Desert in China (25, 26), but these decreased in abundance with increased salinity in alkaline soils of the Yellow River Delta (27, 28). However, there was some commonality in responses across these Chinese regions as bacterial and fungal richness and the abundance of Acidobacteria and Planctomycetes all decreased with higher salinity (25, 27–30). Australian soil microbial communities’ salt tolerance was closely correlated with the salinity levels of the soils they are derived from: here Gammaproteobacteria and Bacteroidetes were associated with increased community-level salt tolerance (31).

There is evidence that microbes may mitigate some of the effects of increased soil salinity by potentially releasing and rebalancing nutrients available for crops. It is possible that these beneficial microbes could be deliberately added to salt-affected soils to help reduce crop yield impacts; for example, one study reported that soil inoculation with *Bacillus aquimaris* rhizobacteria increased the NPK content of wheat leaves under salt stress conditions (32). However, while agricultural bio-inoculation approaches have been suggested many times, they have rarely achieved academic or commercial success due to reduced activation or persistence of the added microbes (33, 34). In this case, any inoculated beneficial microbe(s) would need to be salt-tolerant to be effective.

The theory of microbiome rescue (34) details the possible responses of innate microbial communities to environmental stresses in terms of their structure (the composition of microbial types and abundances) and function—here we define function as supporting crop yield. Under this framework, the possible outcomes of changes in soil salinity levels are as follows: i) microbiomes are innately resistant and show no change in structure or function; ii) microbiomes are innately resilient—salinity initially disturbs microbiome structure and function, but these revert to their original states at some point; iii) there is innate redundancy in microbiomes where salinity induces a change in structure but not function: here, more salt-tolerant taxa increase in abundance and take over and rescue the function of supporting crop yields; iv) elevated salinity permanently changes both structure and function, and crop yields are not supported under elevated salinities. The range of possible microbiome responses will clearly be a function of the size and rate of any salinity changes: gradual changes with slight salinity increases are predicted to result in a different response than large, rapid salinity changes (flooding due to sea wall breaches, for example).

This framework, combined with the empirical work introduced above, leads to the prediction that with sustained periods of elevated low-to-medium levels of salinity irrigation, the operations of natural selection will mean innately more salt-tolerant microbial taxa will become more abundant. The microbiome rescue framework suggests that the reactivation of dormant microbes and the immigration of more tolerant microbes may also contribute to microbiome community structural changes (34). With more sustained exposure to increased salinity, natural selection is predicted to eventually recruit *de novo* mutants that have increased saline tolerance: experimental evolution laboratory work with individual isolates of fungi, bacteria, and nematodes, which are representative of key soil taxa, has shown significant adaptation to increased salinity over ecological timescales of a few hundred generations (35–37). Thus, in the short term and long term, soil microbiomes are predicted to shift in composition due to the sorting effects of salinity: it remains unknown whether altered soil microbiomes can function to support crop yields.

As far as we are aware, the idea of engineering communities using artificial selection (38) has not yet been applied to test if agrifood systems can be primed to better support production under future climate change conditions. The idea that specific interventions can be proactively applied with the aim of pre-adapting agricultural microbiomes to climate change conditions contrasts with the prevailing reactive position of finding remedial interventions once agricultural systems have been damaged.

Here, we test the SoilSalAdapt engineering hypothesis: that due to redundancy in innate soil microbiomes, it is possible to engineer them via saline irrigation to become more tolerant of elevated salinities, and as a result, they can devote more energy to essential soil nutrient cycle processes underpinning soil fertility, and this correspondingly supports crop yields in elevated-salinity environments. We test this hypothesis using mesocosms with three different soil types planted with one of two crops and treated with either freshwater or medium- or high-salinity irrigation. To evaluate this hypothesis, we measured the yield response of crops and changes in soil microbial community structure by DNA amplicon metagenomics and function and fitness by analyzing gene expression and nutrient turnover by amino acid uptake rates.

## RESULTS

### Treatment efficacy

One thousand five hundred liters of three soil types (classified here as sand, silt, and clay, Table S1) was obtained from UK agricultural sites with no previous history of exposure to salinity. A full-factorial randomized blocked design (Fig. S1) comprised 90 30-liter mesocosms (Table S1) planted with either five spinach seeds (*Spinacia oleracea*, Taliabu cultivar) or one Maris Piper seed potato (*Solanum tuberosum*), with five replicates per treatment. Treatments were constructed by diluting the same batch of coastal saline ~30 dS/m agricultural irrigation ditch water with the same rainwater to achieve 3- or 6-dS/m concentrations. These dilutions resulted in differences in sodium, chloride, magnesium, boron, sulfur, potassium, and bicarbonate between irrigation treatments (Table S1, Fig. S1). Mesocosms were drip-irrigated with either rainwater or 3- or 6-dS/m treatments, and reservoirs were agitated weekly and remained ±0.1 dS/m in all cases. All mesocosms also received supplementary fresh rainwater via spray irrigation at levels emulating East of England conditions in 2050, with a 20% reduction in rainfall predicted by the European Environment Agency (Fig. S1; Table S2). All mesocosms underwent an emulated drought (no rain) at the end of the season, with elevated saline pressure by irrigation with 9 dS/m only (constructed by diluting from the same source as the other treatments) for 14 days immediately prior to harvest. All mesocosms were undercover and received the same total volume of water from the same reservoirs (but with different salinity dilutions) across the experiment. The soil salinity of the fresh-, medium-, and high-salinity irrigation treatments averaged 0.08, 0.45, and 0.91 dS/m, respectively, from the May to August irrigation period and increased to 0.62, 1.01, and 1.38 dS/m after 9-dS/m irrigation.

### SoilSalAdapt test 1—effects on soil microbial community structure

Good-quality DNA sequences were obtained for 176 bacterial 16S V3-4 and 185 fungal ITS1 samples from irrigation tanks and soils immediately before and after the emulated drought (Tables S3 and S4). From soils, a total of 21,575,866 bacterial sequence reads clustered into 21,151 > 98% genetic similarity merged-ASVs (mASVs), which approximate species (39), and 21,965,836 fungal reads clustered into 39,801 mASVs. Soil bacterial and fungal communities significantly differed between soil types (*P* < 0.0001), but not crops (other than a weak effect on bacterial abundances *P* = 0.01, which explained just 2% of variance in the data; Table S5) across all richness, types, and abundance biodiversity metrics.

**Fig 1:**
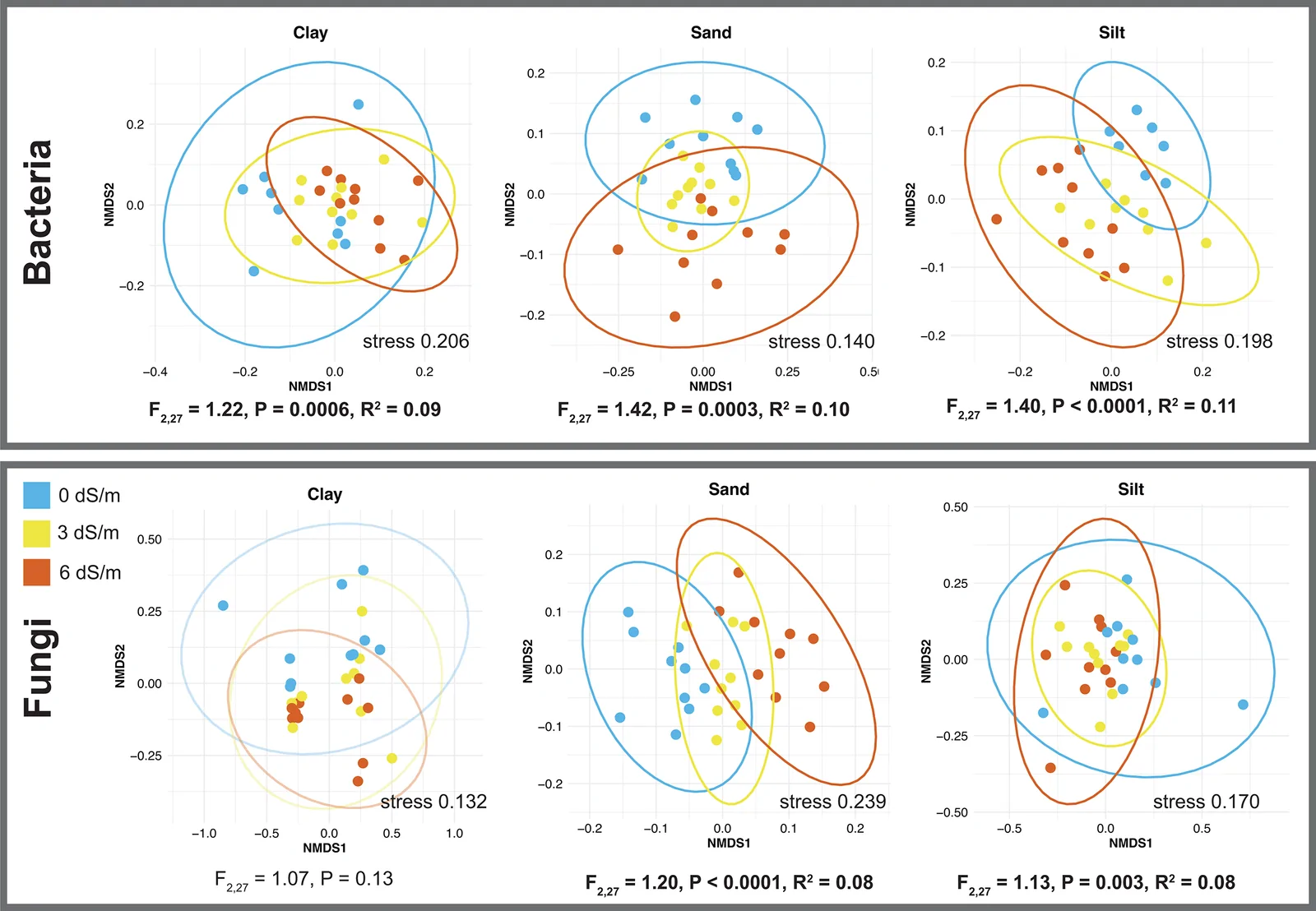
NMDS plots of bacterial and fungal types by irrigation treatment for each soil type before the emulated drought, based on Jaccard dissimilarities with 95% confidence ellipses shown for each treatment. *P*-values were obtained by PermANOVA with 10,000 permutations.

After 4 months, when the variance of microbiomes between soil types was accounted for by blocking permutations within soil types in PermANOVA, analyses reveal there were significant differences in microbiome structures (changes in the types and abundances of bacteria and fungi) between irrigation treatments (*P* = 0.002–0.0001, R_Script1). When each soil type was subsequently analyzed separately (due to the large variance in microbiomes between soil types), irrigation treatment had the greatest and most consistent effect on bacterial community structures: types and abundances differed across all soil types, with an average effect size of 13% with a marked progression in the sorting effect with increasing salinity (Fig. 1; Table S6). The types and abundances of fungi differed between the irrigation treatment in sand and silt soils, but not clay, with an average effect size of 9% (Fig. 1; Table S6). Where a significant effect of irrigation was reported, *post hoc* pairwise comparisons between irrigation treatment levels showed differences in bacterial and fungal types and abundances between most treatments (Table S7).

Taxonomic abundance analyses revealed that bacteria assigned to Rhizobiales (order), including *Geobacillus* (genus) in clay soils and Rhizobiaceae (family) and *Devosia* (genus) in sand soils, became significantly enriched in saline compared to freshwater irrigation treatments (Table S8). Of note, as their names suggest, some members of Rhizobiales are associated with plant rhizospheres and may potentially fix nitrogen and positively affect plant development. Fungi assigned to Mortierellaceae (family) became significantly more abundant, and fungi that are yet to be taxonomically described significantly decreased in sand soil saline irrigation treatments (Table S9). We also focused on key fungal diseases and discovered that mASVs assigned to *Fusarium oxysporum*, *Fusarium venenatum,* and *Alternaria thalapsis*, which cause dry rot/wilt and black spot, significantly decreased in sand soils with potatoes irrigated with 3 and 6 dS/m compared to the freshwater control (*P:* 0.048–0.007, Table S10; Fig. S2).

These observations are in line with the first aspect of the SoilSalAdapt engineering via redundancy hypothesis, predicting microbial communities will diverge between irrigation treatments due to differential levels of saline selection pressure and result in more saline-tolerant taxa becoming enriched (Fig. 1). A significant change in the types of microbial taxa between irrigation treatments is also in line with the aspect of the redundancy mechanism, suggesting the reactivation and selection of dormant microbes may contribute to structural changes. The redundancy mechanism also suggests that immigration of more tolerant taxa may play a role in community structure changes, which here would be from irrigation water. However, there were no mASVs that were in common between irrigation water (recall the same source was diluted for all irrigation treatments) and their respective soils either before or after the emulated drought (see R_Script1). There were some mASVs with higher taxonomic classifications that overlapped between irrigation and soil bacterial communities. For example, mASVs assigned to Rhizobiales (order) comprised ~1% of the 6-dS/m irrigation water microbiome, and mASVs assigned to this family were also significantly enriched in soils irrigated with 6 dS/m (Table S8). However, the identification of similar taxa in irrigation water and those enriched in soils under higher-salinity irrigation treatments does not demonstrate any enriched members derived from irrigation water. The alternative explanation that only innate soil members were selected for and became enriched is equally as likely, given there were 305 mASVs assigned to Rhizobiales in innate soils prior to saline irrigation (from the 0-dS/m treatments prior to 9-dS/m irrigation). Moreover, the presence of specific taxa in irrigation water does not mean these are residents of or adapted to higher salinities as it is likely that members of agricultural soil communities will have been leached from fields into the irrigation ditch from which we sourced the water (Table S1).

Together, these data demonstrate components of the innate soil microbial communities have been engineered by the application of saline irrigation and indicate that potentially beneficial microbes have been enriched and disease agents decreased because of this.

**Fig 2:**
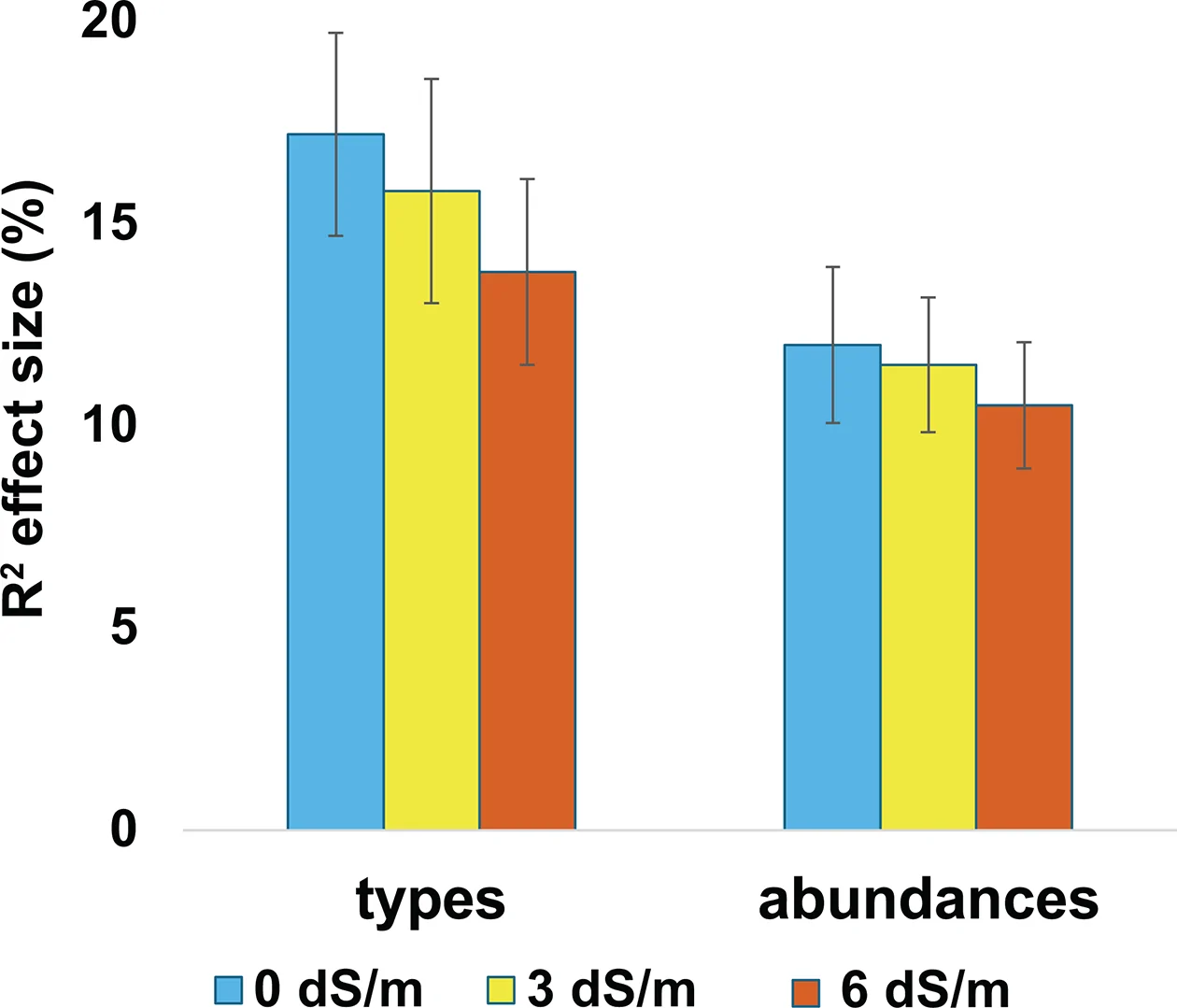
Mean ± SEM magnitudes of significant changes (PermANOVA R^2^ percent effect sizes, Tables S12 and S13) in the types and abundances of soil microbes after exposure to drought with 9-dS/m salinity irrigation in treatments with prior 0-, 3-, or 6-dS/m salinity irrigation.

The types and abundances of taxa in bacterial and fungal communities significantly changed in all treatments after the emulated drought, where soils were irrigated with 9 dS/m, with a substantial list of specific taxa that changed (*P* < 0.0003 by PermANOVAs, Tables S11 to S15). Bacteria were affected twice as much as fungi by 9-dS/m irrigation (from R^2^ values in Tables S12 and S13: types 20% vs 10% and abundances 15% vs 7%, respectively). The overall magnitude of change in microbial communities after 9-dS/m irrigation correlated with pre-drought saline irrigation treatment: the greater the pre-drought saline irrigation concentration, the less the effect of elevated salinity later in the season (Fig. 2). After exposure to 9 dS/m, the mean change in communities irrigated with 6 dS/m was one-fifth less than those irrigated with 0 dS/m. Overall, these data are in line with the adaptive engineering hypothesis: prior irrigation with higher salinities has more strongly sorted and selected for collections of saline-tolerant microbial taxa such that the resulting soil communities are correspondingly affected less by subsequent higher-salinity concentrations due to their greater extents of adaptation to salinity compared to communities with no prior exposure to salinity.

The consistency of soil microbiomes’ responses to saline irrigation can be estimated by evaluating whether similar collections of taxa were selected for with co-occurrence analyses. There were large numbers of significantly (p_adj_ <0.05) co-occurring taxa within treatments (mean 403 and 693 for bacteria and fungi prior to the emulated drought and 3,700 and 731 after, respectively) representing ~18% and 2% of the soil bacterial and fungal microbiomes (Fig. S3 to S5; Tables S16 and S17). Further, acknowledging these proportions are very small, the number of in-common before and after 9-dS/m co-occurring bacterial taxa is significantly greater in the 6-dS/m (mean 1.63%, 95%CI 1.2%–2.15% based on an exact binomial test) than the 0-dS/m (0.39%, 95%CI 0.24%–0.55%) treatment. Overall, this is again in line with the hypothesis predicting irrigation with salinity has sorted for collections of tolerant organisms that underpin soil microbiome adaptation, and some consortia persist when exposed to even greater saline concentrations.

### SoilSalAdapt test 2—effects on soil microbial community function by mRNA analyses

**Fig 3:**
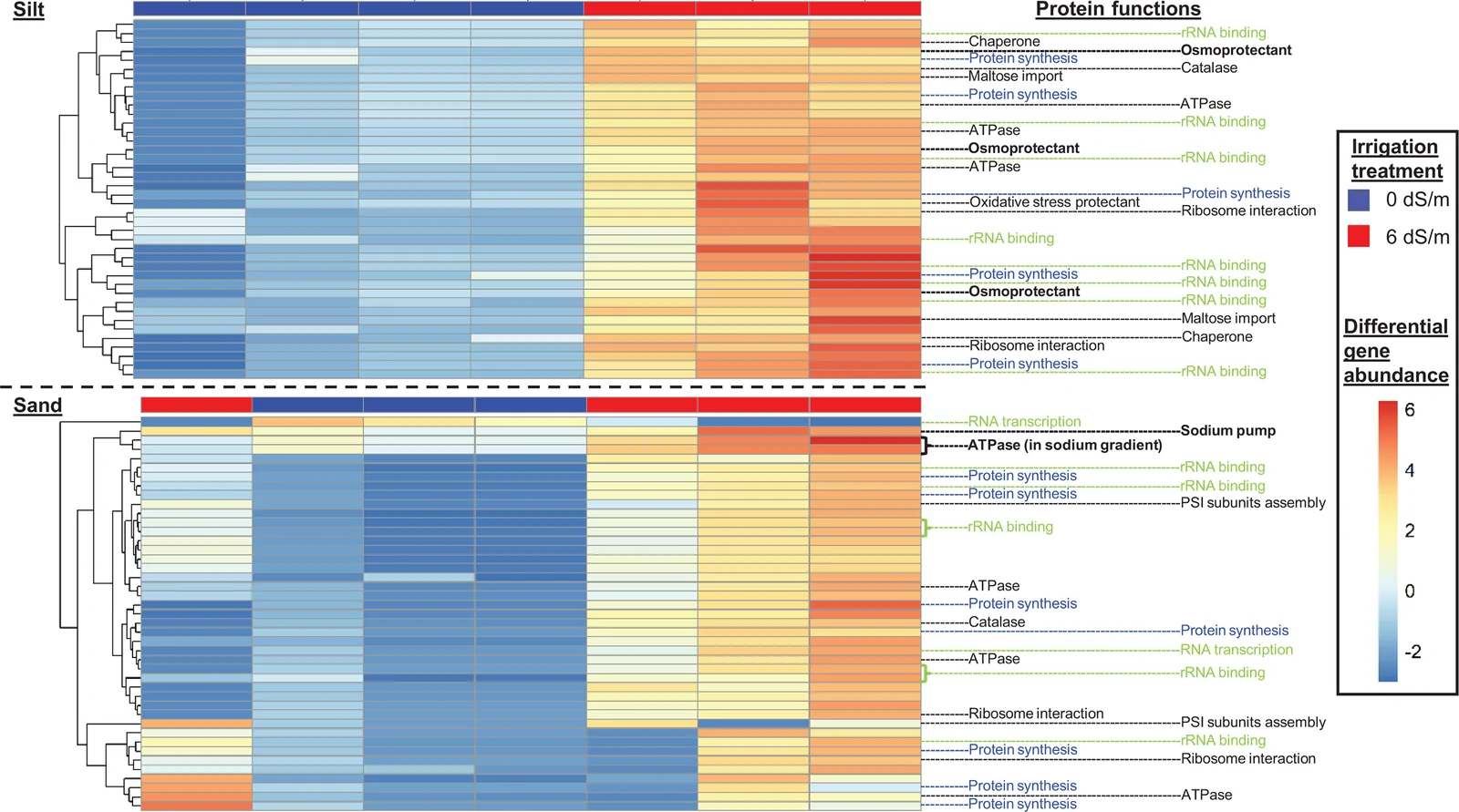
Differential transcript abundances in silt and sand soils. Control (0 dS/m) and saline (6 dS/m) irrigation treatments are represented in columns with blue and red “column headers,” indicating which treatment each column belongs to: see the “Irrigation treatment” legend. The colored cells indicate the extent of differential abundance of each transcript, increasing from yellow to red and decreasing from light to dark blue. Inferred protein functions for transcripts are shown with RNA transcription and rRNA binding in green, protein synthesis in blue, and sodium abundance/processing in bold.

Bacterial and fungal mRNA abundance analysis revealed 40 and 382 genes were significantly differentially expressed (p_adj_ <0.05) between 0- and 6-dS/m treatments for silt and sand soils respectively, and the 40 most differentially expressed genes show clear functional differences (Fig. 3). Many upregulated genes in saline treatments were ascribed to transcripts involved in rRNA binding, protein synthesis, and sodium-related functions (osmoprotection and sodium pumps). We then analyzed genes with sodium-related functions in sand soils, which revealed 11 sodium-related genes with significantly differential expression (p_adj_ <0.05) between 0- and 6-dS/m treatments (Fig. S6), and the majority (6 out of 11) were involved in energy management (ATPases) in sodium gradients. This is in line with the microbiome engineering hypothesis prediction that the operation of selection under saline irrigation has promoted better microbiome energy management under higher salinities.

### SoilSalAdapt test 3—effects on soil microbial community fitness by nutrient turnover

Following Bååth et al. (40), we use relative rates of leucine (an essential amino acid) uptake to evaluate metabolic activity and growth rate (41) and infer nutrient turnover in increasingly saline environments (2 to 20 dS/m). As leucine uptake is also equivalent to growth rate, this also estimates the net fitness of entire soil microbial populations (35). The above transcription data support the prediction that saline irrigation has increased microbes’ ability to devote more energy to nutrient cycling and growth processes under increased salinity. If this is the case, this would correspondingly be manifest as treatments with prior saline irrigation having greater fitness (growth rates) in saline environments. There was a significant difference in microbiome fitness between soil types (*P* = 2 × 10^−5^, ANOVA), so these were analyzed separately. Prior to 9-dS/m irrigation, there was a significant difference in fitness by irrigation treatment in each soil type (*P* < 0.007). However, visual inspection of fitness landscapes (Fig. S7) revealed 0-dS/m clay communities had greater fitness. Conversely, and in line with the SoilSalAdapt hypothesis, the sand and silt soil communities exposed to prior saline irrigation had greater fitness (Fig. S7): e.g., sand communities irrigated with 6 dS/m had twice the fitness of those irrigated with 0 dS/m in an 8-dS/m test environment (Fig. S8). For sand and silt soil communities, this indicates that prior saline irrigation has better adapted these soils’ microbiomes for greater growth and nutrient turnover in higher-salinity environments.

**Fig 4:**
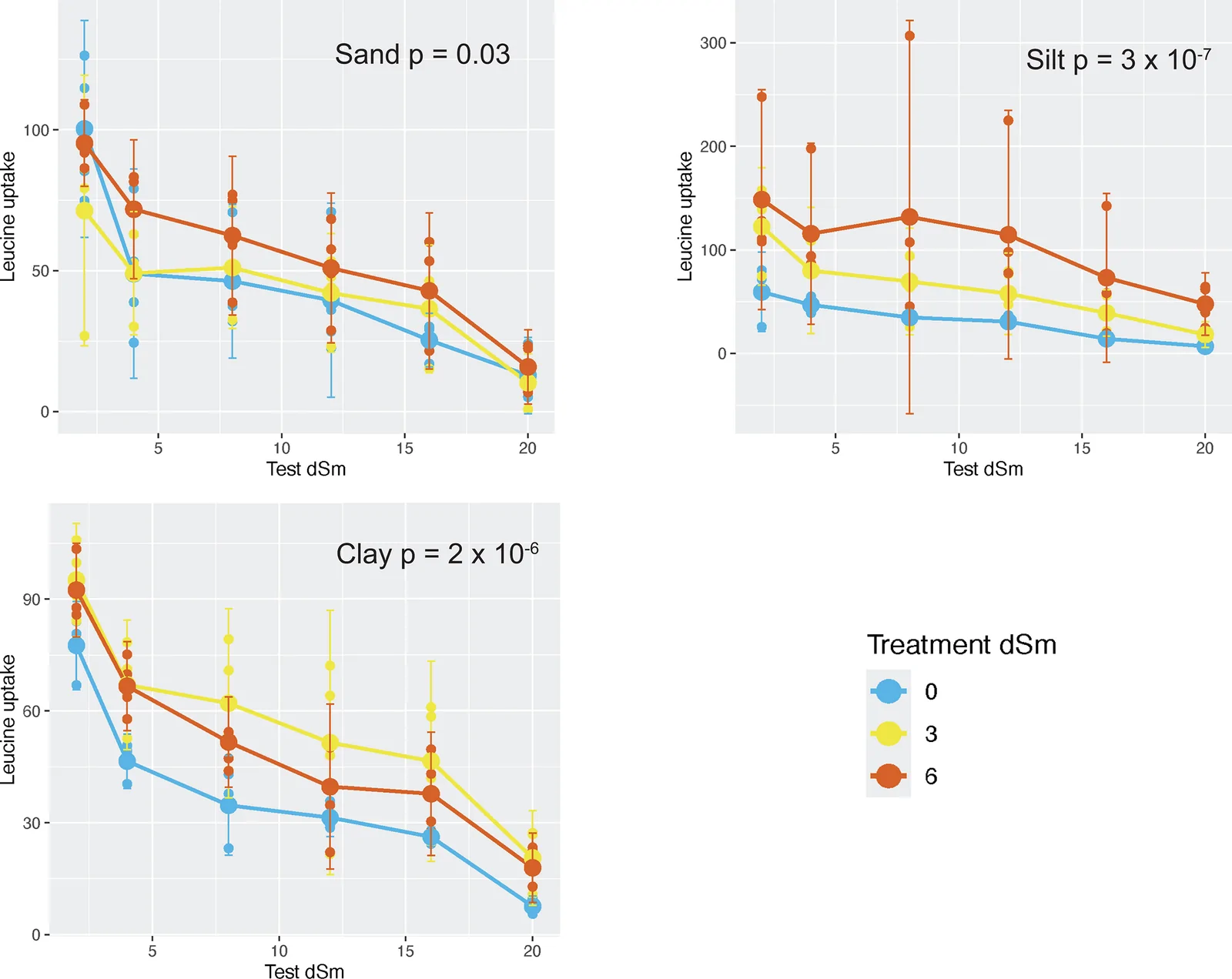
Relative difference in leucine uptake across a 2- to 20-dS/m salinity gradient compared to the 0.1-dS/m control of microbiomes in sand, silt, and clay soils after 9-dS/m irrigation by prior (0, 3, or 6 dS/m) irrigation treatments. Points are expressed as mean ± SEM; *n* = 8, and the probabilities of no differences between linear models with and without dS/m treatment fits are shown.

We also tested the fitness of soil communities after 9-dS/m irrigation as here the sorting effects of increased saline concentrations were greater. The signal for significant fitness differences by prior irrigation treatment was consistent across all soil types (*P* < 0.03), and the treatments with no prior saline irrigation had the lowest fitness in all cases (Fig. 4): this is directly in line with SoilSalAdapt predictions. The fitness landscapes of the sand and silt soil microbiomes show increasing saline tolerance with increasing prior saline irrigation concentration. Thus, prior irrigation with 3 and 6 dS/m has increased the extent of adaptation of soil microbial communities to environments with increased salinities, which allows increased growth and presumably nutrient turnover.

### SoilSalAdapt test 4—effects on crop emergence and yield

The above data and analyses show that saline irrigation has changed soil microbiomes to be better adapted to increased salinity and indicate that they are now able to devote more energy to growth and soil nutrient cycle processes. The prediction arising from this is that this should help support crop yields under elevated-salinity conditions.

**Fig 5:**
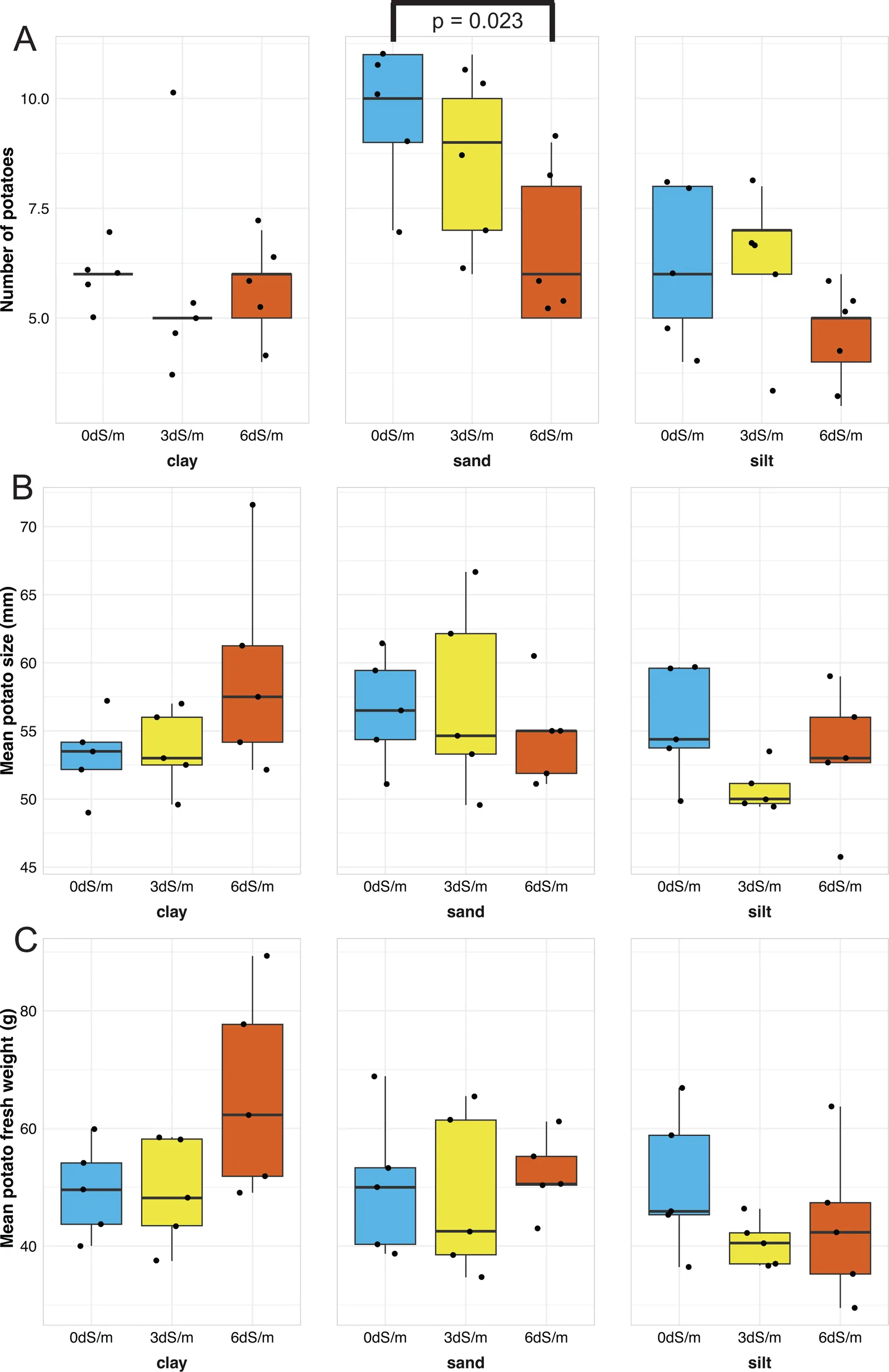
Boxplots of potato yield metrics by irrigation treatment plotted for each soil type with *P* values for the effect of irrigation treatment generated from fits of mixed-effect linear models with irrigation treatment as a fixed and soil type as a random effect (*n* = 5). Only contrasts with *P* < 0.05 are shown. (**A**) The only difference in the number of marketable potatoes per mesocosm between irrigation treatments was reported for sand soils (F_2,36_ = 4.03, *P* = 0.0265), and *post hoc* contrast tests show the only significant difference was between 0- and 6-dS/m treatments (t-ratio = 2.79, 36 df, *P* = 0.0225). Of the potatoes that grew, there were no significant differences in mean potato sizes (**B**); F_2,36_ = 0.62, *P* = 0.54) or mean potato fresh weights (**C**); F_2,36_ = 1.8, *P* = 0.18) between irrigation treatments.

All potato plants emerged and produced above-ground growth (Table S18). The fit of a mixed-effect linear model (with irrigation treatment as a fixed effect and soil type as a random intercept) revealed a significant, though relatively modest, effect of irrigation treatment on the number of marketable potatoes produced overall (F_2,36_ 3.92, *P* = 0.03, R_Script4). However, *post hoc* comparisons of all treatments across all soils revealed the only significant difference in the number of marketable potatoes was between 0 and 6 dS/m in sand soils (t-ratio 2.79, p-adj = 0.023), with *P* > 0.17 for all other within-soil contrasts (R_script4, Fig. 5A). Of the tubers that achieved marketable size, the fit of mixed-effect linear models revealed there was no effect of irrigation treatment on mean potato size (F_2,36_ 0.62, *P* = 0.54, Fig. 5B), mean potato fresh weight (F_2,36_ 1.78, *P* = 0.18, Fig. 5C), or mean dry weight (F_2,36_ = 0.22, *P* = 0.81, Fig. S9, R_Script4).

**Fig 6:**
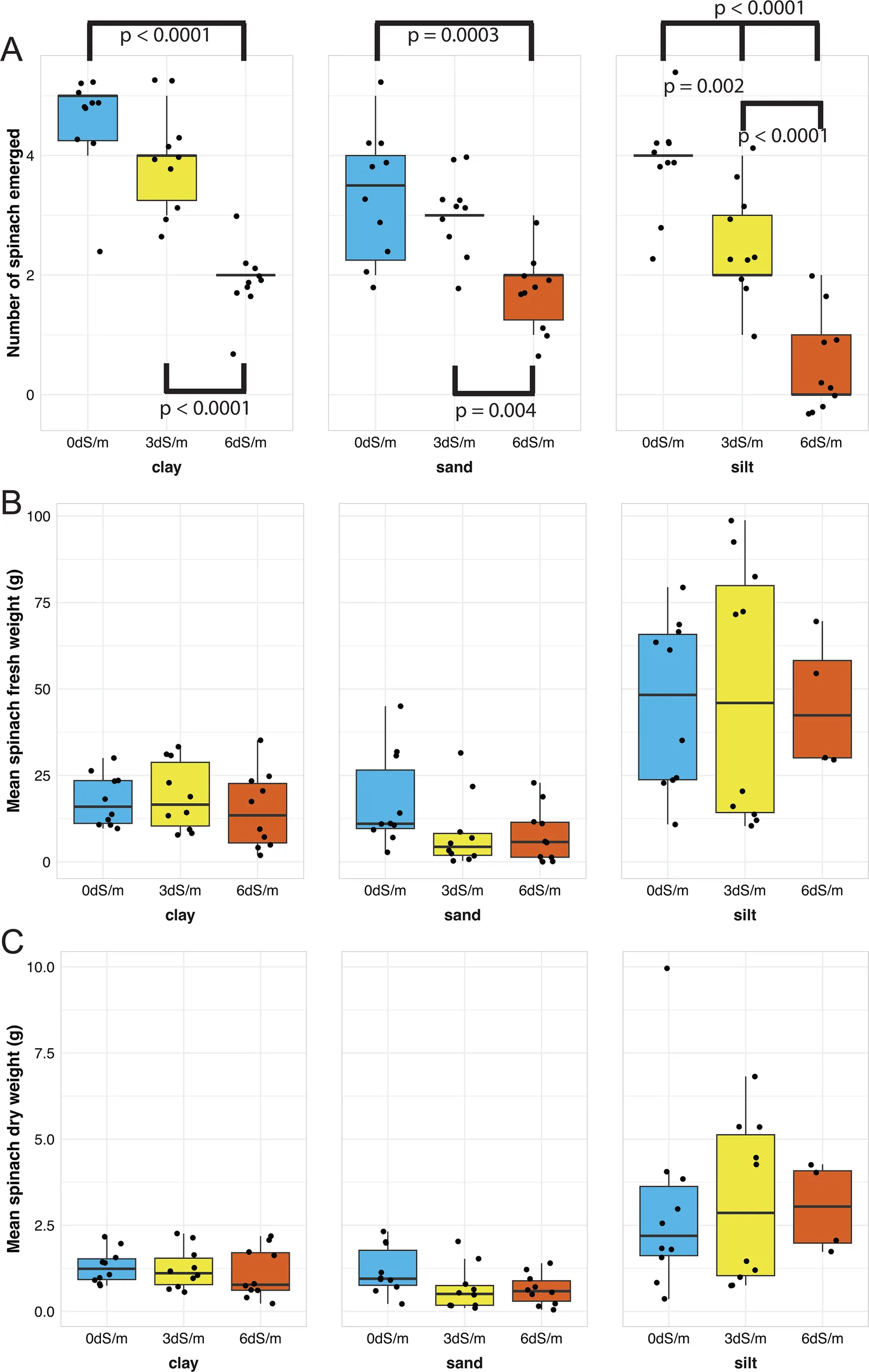
Boxplots of spinach yield metrics by irrigation treatment plotted for each soil type, with *P* values for the effect of irrigation treatment generated from fits of mixed effect linear models with irrigation treatment as a fixed effect and soil type as a random effect (*n* = 5). Only contrasts *P* < 0.05 are shown. (**A**) There was a significant effect of irrigation treatment on spinach emergence overall (F_2,80_ = 67.8, *P* < 2 × 10^−16^), with a significant interaction between treatment and soil type (F_4,80_ = 2.88, *P* = 0.028), and the p-adj values of *post hoc* contrast tests are shown. Of the spinach that grew, there were no significant differences in mean fresh (**B**); F_2,74_ = 2.22, *P* = 0.12) or dry weights (**C**); F_2,74_ = 2.3, *P* = 0.11) between irrigation treatments.

There was a large variance in spinach emergence (Fig. 6A; Table S19), with a mean of 2.8 emerged of five seeds planted per mesocosm. Emergence was significantly affected by irrigation treatment overall (mixed-effect linear model, F_2,80_ = 67.8, *P* < 2 × 10^−16^, R_script4), with a significant but modest interaction with soil type (F_4,80_ = 2.88, *P* = 0.028). There was no clear difference in emergence between soil types (F_2,80_ = 3.1, *P* = 0.055) and no effect of planting time (F_1,80_ = 0.07, *P* = 0.80). Emergence was affected by saline irrigation treatment within each soil type (soil:treatment contrasts *P* < 0.0002). Corrected pairwise *post hoc* contrast tests reveal there was a difference in emergence between 0- and 3-dS/m irrigation in silt soils (t-ratio = 3.55, p_adj_ = 0.002), but no difference in emergence between 0- and 3-dS/m irrigation in clay and sand soils (t-ratios 1.64 and 0.82, and *P*-values 0.24 and 0.69, respectively). There was a significant difference in spinach emergence between 6-dS/m and all other irrigation treatments (p_adj_ <0.004, Fig. 6A) across all soil types. Variance in spinach emergence was controlled for by analyzing the mean weights of emerged spinach plants per mesocosm (Table S19). Linear mixed-effect models reveal that fresh and dry weights did not significantly differ between irrigation treatment overall (F_2, 74_ = 2.2 and 2.3, with *P*-values 0.12 and 0.11 for fresh and dry weights, respectively, Fig. 6B and C), and there was no interaction between irrigation treatment and soil types for these yield parameters (F_4,74_ = 0.90 and 1.23, with *P*-values 0.47 and 0.31, respectively).

Overall, there is evidence that the highest level of saline irrigation (6 dS/m) suppressed the number of marketable potatoes produced in sand soils, but there was no effect of 6 dS/m on potato numbers in clay or silt soils. The emergence of spinach was suppressed by 6-dS/m irrigation across all soils. Moderate levels of saline irrigation (3 dS/m) had no effect on the number of marketable potatoes achieved in any soil and only suppressed spinach emergence in silt soils. However, of the potatoes that reached marketable size and the spinach that emerged, there was no effect of either 3- or 6-dS/m saline irrigation on final potato and spinach fresh and dry weight yield parameters (Fig. 5 and 6).

## DISCUSSION

The data show that the use of low-to-medium levels of saline irrigation changed the structure, function, leucine uptake rates, and fitness of soil microbiomes, with genes involved in osmotolerance and energy regulation upregulated; together, this rendered microbiomes better adapted to elevated salinities. Saline irrigation commenced immediately at planting, and the only significant effect this had on crops was to reduce the numbers of potatoes produced and spinach that emerged. Spinach emergence was strongly suppressed at 6-dS/m irrigation across all soil types but was only suppressed at 3-dS/m irrigation in silt soils; only 6-dS/m irrigation in sand soils suppressed the number of marketable potatoes produced, but saline irrigation had no effect on potato numbers elsewhere. One important observation was the use of 3- and 6-dS/m irrigation treatments and the association between significantly changed soil microbiomes but no significant decrease in spinach or potato fresh or dry weight (of the potatoes and spinach that became established): this implies that the better saline-adapted microbial communities engineered by saline irrigation are equally capable of supporting crop yields. Reduced spinach emergence with elevated salinities is in line with other reports of the suppression of seed emergence with increased salinity (42). This leads to one clear suggestion: to initiate saline irrigation after crops are established. Overall, these observations are in line with microbiome rescue via redundancy theory (34), underpinning the SoilSalAdapt engineering hypothesis. Saline irrigation changed the structure and increased the saline fitness of soil microbiomes, and this likely correspondingly allowed more energy to be dedicated to soil nutrient cycles and of potatoes and spinach that became established since these microbiome changes were not associated with any significant drop in crop yields; these observations correlate with the idea that saline irrigation has engineered the soil microbiome and rescued the function of supporting crop yields under increased salinities.

The dilutions to achieve 3-, 6-, and 9-dS/m irrigation treatments resulted in differences in at least sodium, chloride, magnesium, boron, sulfur, potassium, and bicarbonate between irrigation treatments (Table S1): these dilution differences will have not only been involved in the differential selection pressures to which microbes adapted but also possibly been of differential benefit to crops—and thus also potentially part of yield support—as many of these are also key plant nutrients. The microbiome and crop responses observed between treatments were thus likely a function of differences in all these parameters. The effect of saline irrigation on bacteria was greater than that on fungi, which is in line with other reports (24) and observations of more rapid bacterial responses to other environmental changes (43), and is likely because bacteria have shorter generation times. The data reveal that some rhizosphere-associated beneficial microbes became enriched, potentially aiding crop nutrient uptake under saline irrigation.

The type and magnitude of changes in microbiome structures and crop yields due to saline irrigation differed between soil types, and there are several possibilities that may explain these observations. Differences in soil physical and chemical properties between soil types will have modulated the extent of saline exposure and thus selection pressures realized by microbes in different soils. For example, water will move through porous sand more quickly than clay, and ionic interactions and cation retention will differ across soil types. As shown by the data here and in other studies (39), innate agricultural microbiomes vary significantly between soil types and locations, and correspondingly, different types of microbes were enriched by saline irrigation (Table S5). The enrichment of different types between soils may also have resulted in differences in the types of emerged competitive interactions between microbes in engineered microbiomes. Differences in microbiome structures and interactions of their constituents may have, therefore, resulted in differences in the extent and type of emerged nutrient turnover functions and thus the support of crop yields. It is also feasible that other soil biology (such as invertebrates), and thus the structure and function of the entire soil food webs, will have differentially changed between soil types, also potentially explaining the soil-type differences observed. However, the potential ubiquity of this microbiome engineering approach is indicated by the fact that there were microbiome structural changes with corresponding yield support in all soil types between the freshwater and saline irrigation treatments.

Freshwater is becoming increasingly scarce globally, which is exacerbating competition between agricultural, industrial, and domestic uses (44). Another benefit of this approach is that it would reduce the burden of freshwater use by agriculture. In addition, the observation that DNA assigned to some crop fungal disease agents reduced in saline compared to freshwater treatments tentatively suggests that some crop pathogens are potentially salt-sensitive. This indicates a possible unexpected benefit of saline irrigation: that pesticide inputs could be reduced due to better disease suppression. Perhaps the most appealing aspect of this approach is its simplicity and low cost: most growers at risk of salinity pressures will have irrigation systems in place and have access to saline water. This method simply utilizes the existing infrastructure to engineer innate soils rather than introducing agrochemicals or foreign biological agents, and this will increase the likelihood of uptake.

Overall, this work suggests that growers could start to irrigate with at least 3 (and possibly up to 6) dS/m after crop establishment, with no yield penalty. This will initiate the engineering of soil microbiomes and start to condition soils to be more robust in their support of crop yields in the face of future increasing irrigation, soil salinities, and droughts. Of course, the generality and longevity of this approach need to be evaluated in other crops and soil types with field trials over several years. However, this represents a potentially promising proactive and practical method for preparing food systems for future climate change conditions by engineering innate agricultural soil microbiomes to help support crop yields in a way that will also meet sustainable food security requirements.

## MATERIALS AND METHODS

### Experimental design

One thousand five hundred liters of three soil types (classified as sand, silt, and clay, Table S1) were obtained from UK agricultural sites with no previous history of exposure to salinity. A full-factorial randomized blocked design (Fig. S1) comprised 90 30-liter mesocosms (Table S1) planted with either five spinach seeds (*Spinacia oleracea*, Taliabu cultivar) or one Maris Piper seed potato (*Solanum tuberosum*), with five replicates per treatment. Mesocosms were drip-irrigated with either rainwater or 3- or 6-dS/m treatments constructed by diluting the same batch of coastal saline ~30-dS/m agricultural irrigation ditch water with the same rainwater; reservoirs were agitated weekly and remained ±0.1 dS/m in all cases (Table S1; Fig. S1). All mesocosms also received fresh rainwater via spray irrigation at levels emulating East of England conditions in 2050, with a 20% reduction in rainfall predicted by the European Environment Agency (Fig. S1; Table S2). All mesocosms underwent an emulated drought (no rain) at the end of the season, with elevated saline pressure by irrigation with 9 dS/m only for 14 days immediately prior to harvest. All mesocosms were undercover and received the same total volume of water from the same reservoirs (but with different salinity dilutions) across the experiment. Two consecutive spinach batches were planted and harvested in August and September. The number of emerged plants and total fresh weight were measured per pot. Spinach was dried at 70°C for 48 h and weighed. Potatoes were harvested in September. Tubers above the marketable size of 45 mm were recorded and graded with potato sizing squares (Martin Lishman Ltd) and weighed. Potatoes were then dried at 70°C for 48 h and weighed. No fungicides or other forms of crop protection were applied.

### DNA/RNA extractions, amplification, and sequencing

Five 3-mL topsoil samples were collected before and after 9-dS/m irrigation from each pot and homogenized, and 15 mL of each irrigation water was collected; all were stored at −80°C until R/DNA extraction. Soil samples were dried at 65°C for 24 h before DNA extraction with Qiagen PowerSoil Pro Kits. DNA amplification of the bacterial 16S V3-4 and fungal ITS1 regions with subsequent Illumina sequencing was performed by Eurofins Genomics and Novogene. RNA was extracted from 0- and 6-dS/m treatment wet soils before 9-dS/m irrigation using Qiagen PowerSoil Total RNA Kits. mRNA amplification libraries were depleted to concentrate both bacterial and fungal mRNA from whole RNA extractions and then used to produce cDNA libraries, which were 2 × 150 bp sequenced on an Illumina HiSeq platform by Eurofins Genomics.

### Leucine uptake

Suspensions of free and loosely attached cells from 1 gram of soil (45) were spiked with 10 µL of ³H-leucine (L-[4,5-³H(N)], 37 MBq, 6.734 TBq/mmol) and 10 µL of non-radioactive L-leucine, resulting in final concentrations of ~50 kBq per 1.52 mL and 330 nmol/L, respectively (*n* = 4). Controls were treated with 75 µL of 100% trichloroacetic acid (TCA) prior to kill cells. Uptake treatments were constructed by the addition of NaCl to create conductivities of 0.1, 2, 4, 8, 12, 16, and 20 dS/m. All tubes were incubated at room temperature for 2 h and then treated with TCA to stop activity. Samples were washed following Almas et al. (45), and ³H incorporation was measured using Liquid Scintillation Counting.

### Bioinformatic analyses

#### DNA

Forward reads were quality-filtered and denoised to generate amplicon sequence variants (ASVs) with dada2 (46) using Qiime2 (v.2024.5) (47). Different sequence batches were processed separately and resulting ASV tables combined. Following Fernandez-Huarte et al. (39), the resulting ASVs were clustered into >98% genetic similarity merged-ASVs (mASVs), which approximate species. Bacterial mASVs were taxonomically assigned using the SILVA database (release 138) (48) with 99% identity and fungal mASVs using the Unite database (v.9) (49), with a 97% to 99% dynamic threshold based on accuracy for each fungal lineage using a Bayes classifier in Qiime2. 16S V3-4 and ITS1 taxonomy references and classifiers were generated using ReScript (50). Contaminants from negative controls and mitochondria and chloroplast sequences were filtered out.

#### RNA

Sand and silt soil samples were analyzed separately (clay samples failed), and k-mers counted using Jellyfish (v1.0.3) (51) and Rcorrector (v1.0.7) (52) was used to correct sequencing errors. Adapters were removed with Trimmomatic (v0.39) (53), reads with poor quality removed with FastQC (v0.11.8) (54), and the remainder were used for *de novo* assembly with SPAdes (v3.15.5) (55). Assembly quality was evaluated using rnaQUAST (2.2.4) (56). Open reading frames were extracted with TransDecoder (v5.7.1) (57), and protein similarities against the UniprotKB/Swiss-Prot database were determined with BlastP (v2.15.0) (58). Putative coding regions were predicted using TransDecoder (57) and quantified using Salmon (v1.0.3) (59). Differential gene abundance analysis was performed on all samples using rnaseqGene (v3.19) (60).

### Statistical analyses

Data were analyzed with R (61) with the “tidyverse” (62) library along with the additional libraries detailed below unless stated otherwise. Data were plotted with ggplot2 (part of tidyverse [62]) unless stated otherwise, using color-blind considerate themes. Data and analyses details are available in supplemental code files (R_scripts1-4). Rarefied mASV richness (alpha diversity) was calculated by subsampling to the lowest read depth (32,242 and 55,140 for bacteria and fungi, respectively). Between-sample read depth variance was normalized using within-sample max-min normalization. All data were analyzed under the same standard frequentist statistical framework employing F-distributions to determine the probability that data from different groups were sampled from the same underlying populations. For yield and microbiome richness univariate data, this was achieved by fitting mixed-effects linear models to evaluate the influence of irrigation salinity across crops and soil types. Analyses were conducted in R utilizing the “lme4” package (63) for model fitting and “lmerTest” (64) to obtain *P*-values via the Satterthwaite approximation for denominator degrees of freedom. Irrigation treatment was defined as a fixed effect and soil type as a random effect. This structure allows models to account for the inherent baseline variation in the various responses across different soils and allows the specific impact of saline irrigation to be cleanly isolated. Model assumptions of approximately normal residual distributions were verified through visual inspection of residual QQ plots and Shapiro tests to check for homoscedasticity. Where significant differences by irrigation were detected, the main effects were analyzed from estimated marginal means using the “emmeans” package (65), with subsequent Tukey’s HSD pairwise comparisons with adjustment for multiple testing where appropriate.

All multivariate microbiome ASV data were analyzed via the vegan R package (66) with PermANOVA via the “adonis2” function and “pairwise_adonis2” (67) functions with Jaccard and Ruzicka dissimilarities and 10,000 permutations. This method also uses pseudo-F ratios to calculate *P*-values for main effects and their interactions. The PermANOVA assumption of equal dispersion among groups in multivariate space was tested with betadisper and permutest functions, and the outcomes were corroborated with separate anosim analyses if these were not met. Variance between soils was controlled for with the strata function, which blocked permutation within soil types. Community composition was visualized using non-metric multidimensional scaling (NMDS) based on a Jaccard distance matrix, and a stress value was calculated to assess the goodness-of-fit. To illustrate group dispersion and centroid stability, 95% confidence ellipses were calculated for each treatment level. Differences in specific taxa abundances were tested using “ancombc2” (68) and “aldex.glm” (69). As recommended by Nearing et al. (70), only taxa that overlap between these methods were reported, and differences were visualized with heatmaps. Pairwise correlations between bacterial and fungal co-occurrences were calculated using sparCC in the SCNIC package (71), and correlation networks built with a minimum R and adjusted *P*-value cutoffs of 0.35 and 0.05, respectively. The resulting networks were visualized using Cytoscape (v3.10.2) (72).

## Data Availability

Data and files can be found on FigShare at https://doi.org/10.24385/lincoln.31049746. Nucleic acid sequence data are available in the NCBI Sequence Read Archive (SRA) under BioProject number PRJNA1270703.

## SUPPLEMENTAL MATERIAL

Supplemental material aem.00437-26-s0001.docx Fig. S1 to S9; Tables S1 to S19.

ASM does not own the copyrights to Supplemental Material that may be linked to, or accessed through, an article. The authors have granted ASM a non-exclusive, world-wide license to publish the Supplemental Material files. Please contact the corresponding author directly for reuse.
