# Supplementary material for: Supporting crop yields under climate change by engineering innate soil microbiomes
Source: Appl Environ Microbiol. 2026 Jul 13;92(8):e00437-26. doi: 10.1128/aem.00437-26 (PMC13488346; doi:10.1128/aem.00437-26)
Supplement: Supplementary file 1 [file aem.00437-26-s0001.docx]

**Supplementary Information**

**Supplementary Figures**

A

B

**Figure S1**. A) Experimental set-up. Each circle represents a sample mesocosm (pot), with the inside colour representing the crop (green = spinach; orange = potato) and the outside colour representing irrigation treatment. The coloured rectangles and lines represent the three irrigation treatments tested: 0 dS/m, 3 dS/m and 6 dS/m. Crop and irrigation treatments are randomised within each soil type block. B) Saline irrigation treatments were applied via a drip ring and fresh water emulating rainfall by a spray nosel (purple). See Table S2 for rates.

**Figure S2.** Differences in relative abundance (+/- SEM) of Alternaria and Fusarium species for potatoes (A) and spinach (B) between 0, 3, and 6 dS/m irrigation treatments. p-values were obtained by PermANOVA**.** Significant differences p_adj_ < 0.05 are in bold.

After 9 dS/m - spinach

Before 9 dS/m - spinach

After 9 dS/m - potatoes

Before 9 dS/m - potatoes

**Figure S3**. Co-occurrence networks of bacterial and fungal genera in silt before (A & B) and after 9dS/m (C & D). Blue nodes represent bacterial taxa, and red nodes represent fungal taxa. Green edges indicate a positive and purple a negative co-occurrence.

Before 9 dS/m - potatoes

After 9 dS/m - potatoes

Before 9 dS/m - spinach

After 9 dS/m - spinach

**Figure S4**. Co-occurrence networks of bacterial and fungal genera in clay before (A & B) and after 9dS/m (C & D). Blue nodes represent bacterial taxa, and red nodes represent fungal taxa. Green edges indicate a positive and purple a negative co-occurrence.

After 9 dS/m - spinach

After 9 dS/m - potatoes

Before 9 dS/m - spinach

Before 9 dS/m - potatoes

**Figure S5**. Co-occurrence networks of bacterial and fungal genera in sand before (A & B) and after 9dS/m (C & D). Blue nodes represent bacterial taxa, and red nodes represent fungal taxa. Green edges indicate a positive and purple a negative co-occurrence.

**Figure S6**. Differential transcript abundance analysis for sodium related genes in sand.

Control (0 dS/m) and saline (6 dS/m) irrigation treatments are in columns with blue and red ‘column headers’ indicating which treatment each column belongs to: see the “Irrigation treatment’ legend. The coloured cells in the figure represent the differential abundance of each gene in each sample, with an increase in abundance from yellow to red, and a decrease in abundance from light blue to dark blue. The text on the right of the figures represents the known function of the protein for each gene.

**Figure S7**. Relative difference in leucine uptake across a 2 to 20 dS/m salinity gradient (compared to the 0.1 dS/m control) of microbiomes in sand, silt and clay soils before 9 dS/m irrigation by prior (0, 3 or 6 dS/m) irrigation treatments. Significance of difference between treatments are from comparisons of linear models with and without dS/m treatments included; see Script3_Leucine_analyses.R.

**Figure S8.** Relative difference in leucine uptake in an 8 dS/m salinity environment of microbiomes in sand soils by prior 0, 3 or 6 dS/m irrigation treatments. Leucine uptake differs by treatment (p = 0.049, Kruskal-Wallis test)

**Figure S9**. Boxplot of mean potato dry weight per mesocosm by irrigation treatment. ANOVA on the fit of a mixed effect linear model, with soil as a random and treatment a fixed effect, reveal there was no effect of irrigation treatment on potato dry weight (F_2,36_ = 0.22, p = 0.81, R_Script4)

**Supplementary Tables**

**Table S1.** Physical and chemical soil and water properties. Analyses by LanCrop Laboratories (<https://www.lancrop.com/>). Saline irrigation treatments were achieved by diluting the same batch of coastal saline ~30 dS/m agricultural irrigation ditch water sourced from Fosdyke Wash in Lincolnshire (52°53'44.1"N 0°04'11.2"E; V3W9+6WJ Spalding) with the same rainwater tabled here.

| **Parameter** | **Soil classification** | | | **Irrigation water** | | | |
| --- | --- | --- | --- | --- | --- | --- | --- |
|  | **SAND** | **SILT** | **CLAY** | **Rain** | **3dS/m** | **6dS/m** | **9dS/m** |
| Silt (%) | 49.47 | 50.46 | 46.09 |  |  |  |  |
| Clay (%) | 11.38 | 11.14 | 17.09 |  |  |  |  |
| Sand (%) | 39.15 | 38.4 | 36.82 |  |  |  |  |
| Calcium (ppm) | 3279 | 4585 | 4144 | 5 | 23 | 40 | 55 |
| Magnesium (ppm) | 87 | 79 | 74 | 1 | 61 | 127 | 194 |
| Manganese (ppm) | 48 | 40 | 58 | 0.02 | 0.03 | < 0.01 | 0.02 |
| Boron (ppm) | 1.58 | 4.31 | 2.21 | 0.02 | 0.27 | 0.54 | 0.80 |
| Copper (ppm) | 11.4 | 7.8 | 6.6 | 0.01 | 0.01 | 0.01 | 0.01 |
| Molybdenum (ppm) | 0.02 | 0.03 | 0.03 | < 0.01 | < 0.01 | < 0.01 | < 0.01 |
| Iron (ppm) | 738 | 1406 | 1072 | < 0.1 | < 0.1 | < 0.1 | < 0.1 |
| Zinc (ppm) | 14.2 | 8.7 | 7.3 | 0.25 | 0.74 | 0.25 | 0.35 |
| Sulphur (ppm) | 7 | 14 | 16 | 1 | 46 | 89 | 134 |
| Phosphorus (ppm) | 60 | 91 | 19 | 0.04 | 0.10 | 0.06 | 0.15 |
| Potassium (ppm) | 224 | 375 | 132 | < 2 | 24 | 51 | 78 |
| Sodium (ppm) | 15 | 30 | 37 | 3 | 518 | > 625 | > 625 |
| Org. Matter DUMAS (%) | 2.4 | 2.6 | 3.1 | - | - | - | - |
| pH | 7 | 7.9 | 7.5 | 6.4 | 6.3 | 7.0 | 6.8 |
| C.E.C. (meq/100g) | 20.3 | 25 | 22 | - | - | - | - |
| **dS/m (mmhos/cm)** | - | - | - | **0.06** | **2.97** | **5.99** | **8.87** |
| Chloride (mg/l) | - | - | - | 8.6 | 934.9 | > 1250 | > 1250 |
| Nitrate N (mg/l) | - | - | - | 2.13 | 2.93 | 1.09 | 2.58 |
| Ammonium N (mg/l) | - | - | - | 0.93 | 0.46 | 0.37 | 0.42 |
| Bicarbonate (mg/l) | - | - | - | 6 | 35 | 78 | 102 |

**Table S2.** Irrigation and emulated rainfall volumes per month for the mesocosms, and temperatures recorded inside the polytunnel. Emulated rainfall rates are from the UK Met Office monthly precipitation for East Anglia with a 20% reduction in line with predicted rainfall rates from the European Environment Agency for 2071-2100 <https://www.eea.europa.eu/en/analysis/maps-and-charts/projected-changes-in-annual-and-6>. Irrigation was adjusted to combat heat stress which was particularly high in July and August in the UK: while mean ambient (outside polytunnel) temperatures in July and August 2022 were 21°C there was a heatwave when the ambient temperature achieved 40°C. Indeed, the highest temperature ever recorded in England of 40.3°C was on July 19, 2022, at Coningsby, Lincolnshire – just 30 km (20 miles) from the experimental site; see https://www.metoffice.gov.uk/research/climate/maps-and-data/uk-climate-extremes. Also, July 2022 was the driest July since 1935 in the UK; see <https://www.metoffice.gov.uk/about-us/news-and-media/media-centre/weather-and-climate-news/2022/driest-july-in-england-since-1935>. In many respects this season naturally emulated future climate change conditions just as our experiment intended to do.

|  | May | June | July | August | September |
| --- | --- | --- | --- | --- | --- |
| Rainwater (mm) | 0 | 24 | 14 | 5 | 0 |
| Irrigation (mm) | 22 | 23 | 197 | 148 | 10 |
| Mean temp (°C) | NA | NA | 26 | 25 | 23 |

**Table S3**. Details of soil 16S and ITS DNA sequence information; number of reads are those taken forward for analyses. Data are also available in the associated file ‘ASV read numbers.csv’.

| **Mesocosm** | **Treatment** | **Soil** | **16S (Bacteria)** | | | | **ITS (Fungi)** | | | |
| --- | --- | --- | --- | --- | --- | --- | --- | --- | --- | --- |
|  |  |  | **Before 9dS/m** | | **After 9dS/m** | | **Before 9dS/m** | | **After 9dS/m** | |
|  |  |  | **Sequenced** | **No. reads** | **Sequenced** | **No. reads** | **Sequenced** | **No. reads** | **Sequenced** | **No. reads** |
| 1 | 3dS/m | Silt | Yes | 117,361 | Yes | 181,366 | Yes | 191,014 | Yes | 55,140 |
| 2 | 3dS/m | Silt | Yes | 102,577 | Yes | 177,261 | Yes | 190,895 | Yes | 94,404 |
| 3 | 0dS/m | Silt | Yes | 88,069 | Yes | 182,629 | Yes | 366,092 | Yes | 111,391 |
| 4 | 6dS/m | Silt | Yes | 82,469 | Yes | 189,390 | Yes | 374,407 | Yes | 81,667 |
| 5 | 6dS/m | Silt | Yes | 132,428 | Yes | 93,670 | Yes | 364,002 | Yes | 105,534 |
| 6 | 3dS/m | Silt | Yes | 110,242 | Yes | 181,302 | Yes | 127,148 | Yes | 162,091 |
| 7 | 0dS/m | Silt | Yes | 115,155 | Yes | 189,549 | Yes | 173,721 | Yes | 96,679 |
| 8 | 3dS/m | Silt | Yes | 94,373 | Yes | 176,953 | Yes | 211,902 | Yes | 74,214 |
| 9 | 3dS/m | Silt | Yes | 91,600 | Yes | 160,240 | Yes | 368,973 | Yes | 128,342 |
| 10 | 6dS/m | Silt | Yes | 103,757 | Yes | 174,070 | Yes | 362,561 | Yes | 95,617 |
| 11 | 0dS/m | Silt | Yes | 112,489 | Yes | 178,985 | Yes | 325,404 | Yes | 90,229 |
| 12 | 6dS/m | Silt | Yes | 102,663 | Yes | 175,430 | Yes | 170,886 | Yes | 81,431 |
| 13 | 3dS/m | Silt | Yes | 95,306 | Yes | 82,797 | Yes | 166,970 | Yes | 89,813 |
| 14 | 0dS/m | Silt | Yes | 98,332 | Yes | 172,898 | Yes | 152,108 | Yes | 97,300 |
| 15 | 0dS/m | Silt | Yes | 95,341 | Yes | 180,740 | Yes | 132,533 | Yes | 79,635 |
| 16 | 6dS/m | Silt | Yes | 90,154 | Yes | 177,446 | Yes | 76,385 | Yes | 90,486 |
| 17 | 0dS/m | Silt | Yes | 90,895 | Yes | 174,342 | Yes | 161,189 | Yes | 79,827 |
| 18 | 3dS/m | Silt | Yes | 60,976 | Yes | 174,965 | Yes | 180,320 | Yes | 104,222 |
| 19 | 6dS/m | Silt | Yes | 92,701 | Yes | 179,427 | Yes | 98,903 | No | - |
| 20 | 0dS/m | Silt | Yes | 90,830 | Yes | 179,164 | Yes | 184,936 | Yes | 123,889 |
| 21 | 3dS/m | Silt | No | - | Yes | 181,370 | Yes | 184,908 | Yes | 111,467 |
| 22 | 6dS/m | Silt | Yes | 110,607 | Yes | 108,485 | Yes | 188,490 | Yes | 68,472 |
| 23 | 6dS/m | Silt | Yes | 82,266 | Yes | 64,683 | Yes | 167,223 | Yes | 81,937 |
| 24 | 6dS/m | Silt | Yes | 103,615 | Yes | 49,952 | Yes | 189,541 | Yes | 78,326 |
| 25 | 3dS/m | Silt | Yes | 101,787 | Yes | 49,408 | Yes | 181,618 | Yes | 84,564 |
| 26 | 3dS/m | Silt | Yes | 78,780 | Yes | 80,150 | Yes | 186,879 | Yes | 116,725 |
| 27 | 6dS/m | Silt | Yes | 94,715 | Yes | 48,515 | Yes | 174,289 | Yes | 119,884 |
| 28 | 0dS/m | Silt | No | - | Yes | 68,276 | Yes | 195,841 | Yes | 112,216 |
| 29 | 0dS/m | Silt | No | - | Yes | 82,461 | Yes | 196,533 | Yes | 88,056 |
| 30 | 0dS/m | Silt | No | - | Yes | 73,487 | Yes | 77,289 | Yes | 130,419 |
| 31 | 3dS/m | Clay | Yes | 145,447 | Yes | 119,960 | Yes | 182,481 | Yes | 154,503 |
| 32 | 3dS/m | Clay | Yes | 183,130 | Yes | 105,800 | Yes | 179,092 | Yes | 85,118 |
| 33 | 0dS/m | Clay | Yes | 178,155 | Yes | 33,095 | Yes | 181,790 | Yes | 83,594 |
| 34 | 6dS/m | Clay | Yes | 172,028 | Yes | 104,390 | Yes | 180,297 | Yes | 90,388 |
| 35 | 6dS/m | Clay | Yes | 169,815 | Yes | 81,653 | Yes | 187,612 | Yes | 79,235 |
| 36 | 3dS/m | Clay | Yes | 167,330 | Yes | 54,106 | Yes | 172,510 | Yes | 92,498 |
| 37 | 0dS/m | Clay | No | - | Yes | 97,889 | Yes | 169,829 | Yes | 95,066 |
| 38 | 3dS/m | Clay | Yes | 168,680 | Yes | 51,173 | Yes | 173,033 | Yes | 128,477 |
| 39 | 3dS/m | Clay | Yes | 175,479 | Yes | 32,242 | Yes | 179,369 | Yes | 92,358 |
| 40 | 6dS/m | Clay | Yes | 179,112 | Yes | 99,248 | Yes | 176,420 | Yes | 73,860 |
| 41 | 0dS/m | Clay | Yes | 174,166 | Yes | 49,416 | Yes | 179,297 | Yes | 98,385 |
| 42 | 6dS/m | Clay | Yes | 174,948 | Yes | 87,483 | Yes | 175,608 | Yes | 71,772 |
| 43 | 3dS/m | Clay | Yes | 126,860 | Yes | 45,317 | Yes | 176,558 | Yes | 98,404 |
| 44 | 0dS/m | Clay | Yes | 178,193 | Yes | 56,993 | Yes | 179,710 | Yes | 119,433 |
| 45 | 0dS/m | Clay | Yes | 170,692 | Yes | 54,735 | Yes | 167,048 | Yes | 79,956 |
| 46 | 6dS/m | Clay | Yes | 175,299 | Yes | 58,584 | Yes | 174,830 | Yes | 80,519 |
| 47 | 0dS/m | Clay | Yes | 190,833 | Yes | 54,792 | Yes | 106,199 | Yes | 104,694 |
| 48 | 3dS/m | Clay | Yes | 178,167 | Yes | 39,873 | Yes | 145,566 | Yes | 93,813 |
| 49 | 6dS/m | Clay | Yes | 172,861 | Yes | 83,753 | Yes | 69,262 | Yes | 91,635 |
| 50 | 0dS/m | Clay | Yes | 128,864 | Yes | 46,272 | Yes | 92,861 | Yes | 115,903 |
| 51 | 3dS/m | Clay | Yes | 177,418 | Yes | 89,645 | Yes | 81,763 | Yes | 160,193 |
| 52 | 6dS/m | Clay | Yes | 183,458 | Yes | 55,162 | Yes | 71,826 | Yes | 88,006 |
| 53 | 6dS/m | Clay | Yes | 187,204 | Yes | 92,824 | Yes | 123,617 | Yes | 97,007 |
| 54 | 6dS/m | Clay | Yes | 184,585 | Yes | 62,869 | Yes | 123,698 | Yes | 70,084 |
| 55 | 3dS/m | Clay | Yes | 178,876 | Yes | 78,585 | Yes | 76,652 | Yes | 83,077 |
| 56 | 3dS/m | Clay | Yes | 178,607 | Yes | 67,186 | Yes | 96,698 | Yes | 105,061 |
| 57 | 6dS/m | Clay | Yes | 176,076 | Yes | 93,118 | Yes | 99,806 | Yes | 104,972 |
| 58 | 0dS/m | Clay | Yes | 173,815 | Yes | 88,130 | Yes | 117,496 | Yes | 117,991 |
| 59 | 0dS/m | Clay | Yes | 175,741 | Yes | 52,423 | Yes | 62,036 | Yes | 112,382 |
| 60 | 0dS/m | Clay | Yes | 187,814 | Yes | 77,257 | Yes | 113,673 | Yes | 70,643 |
| 61 | 3dS/m | Sand | Yes | 182,774 | Yes | 43,637 | Yes | 73,101 | Yes | 80,521 |
| 62 | 3dS/m | Sand | Yes | 175,740 | Yes | 46,678 | Yes | 72,632 | Yes | 104,160 |
| 63 | 0dS/m | Sand | Yes | 183,824 | Yes | 90,518 | Yes | 138,635 | Yes | 104,908 |
| 64 | 6dS/m | Sand | Yes | 178,756 | Yes | 86,952 | Yes | 84,179 | Yes | 102,086 |
| 65 | 6dS/m | Sand | Yes | 121,033 | Yes | 68,779 | Yes | 89,126 | Yes | 88,022 |
| 66 | 3dS/m | Sand | Yes | 186,551 | Yes | 62,473 | Yes | 80,157 | Yes | 84,732 |
| 67 | 0dS/m | Sand | Yes | 155,923 | Yes | 61,200 | Yes | 65,735 | Yes | 106,274 |
| 68 | 3dS/m | Sand | Yes | 195,592 | Yes | 75,270 | Yes | 74,406 | Yes | 72,814 |
| 69 | 3dS/m | Sand | Yes | 185,402 | Yes | 103,156 | Yes | 91,137 | Yes | 75,161 |
| 70 | 6dS/m | Sand | Yes | 163,928 | Yes | 49,785 | Yes | 60,491 | Yes | 65,504 |
| 71 | 0dS/m | Sand | Yes | 179,930 | Yes | 48,645 | Yes | 62,778 | Yes | 75,003 |
| 72 | 6dS/m | Sand | Yes | 172,780 | Yes | 100,431 | Yes | 65,369 | Yes | 85,278 |
| 73 | 3dS/m | Sand | Yes | 180,619 | Yes | 84,629 | Yes | 77,520 | Yes | 84,166 |
| 74 | 0dS/m | Sand | Yes | 179,540 | Yes | 65,094 | Yes | 71,483 | Yes | 68,902 |
| 75 | 0dS/m | Sand | Yes | 179,143 | Yes | 92,931 | Yes | 72,214 | Yes | 85,410 |
| 76 | 6dS/m | Sand | Yes | 182,768 | Yes | 146,440 | Yes | 105,802 | Yes | 91,841 |
| 77 | 0dS/m | Sand | Yes | 177,941 | No | - | Yes | 85,396 | No | - |
| 78 | 3dS/m | Sand | Yes | 179,967 | Yes | 48,767 | Yes | 76,912 | Yes | 99,932 |
| 79 | 6dS/m | Sand | Yes | 169,099 | Yes | 52,220 | Yes | 86,105 | Yes | 89,006 |
| 80 | 0dS/m | Sand | Yes | 186,850 | Yes | 50,723 | Yes | 142,034 | Yes | 78,728 |
| 81 | 3dS/m | Sand | Yes | 177,479 | Yes | 79,296 | Yes | 105,577 | Yes | 96,342 |
| 82 | 6dS/m | Sand | Yes | 174,905 | Yes | 74,839 | Yes | 122,792 | Yes | 95,816 |
| 83 | 6dS/m | Sand | Yes | 176,344 | Yes | 67,530 | Yes | 70,976 | Yes | 107,215 |
| 84 | 6dS/m | Sand | Yes | 179,448 | Yes | 84,830 | Yes | 75,845 | Yes | 89,653 |
| 85 | 3dS/m | Sand | Yes | 176,049 | Yes | 93,495 | Yes | 82,657 | Yes | 75,933 |
| 86 | 3dS/m | Sand | Yes | 180,633 | Yes | 76,583 | Yes | 95,146 | Yes | 85,789 |
| 87 | 6dS/m | Sand | Yes | 179,819 | Yes | 50,680 | Yes | 119,386 | Yes | 58,164 |
| 88 | 0dS/m | Sand | Yes | 174,707 | No | - | Yes | 112,125 | No | - |
| 89 | 0dS/m | Sand | Yes | 132,316 | Yes | 122,266 | Yes | 86,710 | Yes | 85,409 |
| 90 | 0dS/m | Sand | Yes | 179,016 | Yes | 50,047 | Yes | 79,789 | Yes | 78,248 |

**Table S4**. Details of irrigation water 16S and ITS DNA sequence information; number of reads are those taken forward for analyses.

Analyses underlying the statistical outputs for tables S5-S7 and S11-S12 are in ‘Script1_Microbiome_structure_analyses.R’

**Table S5.** Effects of soil and crop type on bacterial and fungal mesocosm communities before and after the emulated drought. Analyses of rarefied richness by ANOVA on fitted linear models. Analyses of min-max scaled normalized data by PermANOVA (adonis2 formula = norm_ASVs ~ Soil + Crop) on Jaccard and Ruzicka dissimilarities for types and abundance data. Significant differences p < 0.05 are in bold; eta^2^ and R^2^ effect sizes are shown where tests report significance.

|  | Bacterial 16S | | | Fungal ITS | | |
| --- | --- | --- | --- | --- | --- | --- |
|  | Numbers | Types | Abundances | Numbers | Types | Abundances |
| Soil - before | **p = 1x10^-7^, eta^2^ = 0.33** | **p < 1x10^-4^, R^2^ = 0.34** | **p < 1x10^-4^, R^2^ = 0.40** | **p = 2x10^-16^, eta^2^ = 0.61** | **p < 1x10^-4^, R^2^ = 0.22** | **p < 1x10^-4^, R^2^ = 0.40** |
| Crop - before | p = 0.31 | p = 0.11 | **p = 0.01,**  **R^2^ = 0.02** | p = 0.09 | p = 0.20 | p = 0.26 |
| Soil - after | **p = 1x10^-10^, eta^2^ = 0.48** | **p < 1x10^-4^, R^2^ = 0.23** | **p < 1x10^-4^, R^2^ = 0.23** | **p = 1x10^-7^, eta^2^ = 0.35** | **p < 1x10^-4^, R^2^ = 0.08** | **p < 1x10^-4^, R^2^ = 0.09** |

**Table S6.** Effects of irrigation treatment on bacterial and fungal mesocosm communities prior to the emulated drought. Analyses of rarefied richness by ANOVA on linear model fits. Analyses of min-max scaled normalized data by PermANOVA on Jaccard and Ruzicka dissimilarities for types and abundance data. Significant differences p < 0.05 are in bold; eta^2^ and R^2^ effect sizes are shown where tests report significance.

|  | Bacteria 16S | | | Fungi ITS | | |
| --- | --- | --- | --- | --- | --- | --- |
|  | Numbers | Types | Abundances | Numbers | Types | Abundances |
| Sand | p = 0.55 | **p = 3x10^-4^, R^2^ = 0.10** | **p = 7x10^-4^, R^2^ = 0.15** | p = 0.30 | **p < 1x10^-4^, R^2^ = 0.08** | **p = 0.007, R^2^ = 0.10** |
| Silt | **p = 0.026, eta^2^ = 0.27** | **p < 1x10^-4^, R^2^ = 0.11** | **p = 2x10^-4^, R^2^ = 0.20** | p = 0.67 | **p = 0.0034,**  **R^2^ = 0.08** | **p = 0.026,**  **R^2^ = 0.10** |
| Clay | p = 0.56 | **p = 6x10^-4^, R^2^ = 0.09** | **p = 8x10^-4^,**  **R^2^ = 0.15** | p = 0.28 | p = 0.13 | p = 0.22 |

**Table S7.** Pairwise post-hoc PermANOVA tests where the effect of irrigation treatment on microbial types and abundances reported in Table S6 was significant. Significant differences p < 0.05 are in bold; R^2^ effect sizes are shown where tests report significance.

|  | dS/m comparison | Bacteria | | Fungi | |
| --- | --- | --- | --- | --- | --- |
| Pairwise.adonis2 tests |  | Types | Abundance | Types | Abundance |
| Sand | 0 v 3 | **p = 0.0118**  **R^2^ = 0.07** | **p = 0.0237**  **R^2^ = 0.11** | **p = 0.0082**  **R^2^ = 0.06** | p = 0.06 |
|  | 3 v 6 | **p = 0.0235**  **R^2^ = 0.07** | **p = 0.0046**  **R^2^ = 0.12** | **p = 0.0091**  **R^2^ = 0.05** | p = 0.24 |
|  | 0 v 6 | **p = 0.0007**  **R^2^ = 0.08** | **p = 0.0091**  **R^2^ = 0.12** | **p < 1^10-4**  **R^2^ = 0.07** | **p = 0.0041**  **R^2^ = 0.09** |
| Silt | 0 v 3 | **p = 0.0006**  **R^2^ = 0.08** | **p = 0.0108**  **R^2^ = 0.13** | **p = 0.042**  **R^2^ = 0.06** | **p = 0.020**  **R^2^ = 0.08** |
|  | 3 v 6 | **p = 0.0112**  **R^2^ = 0.07** | p = 0.08 | p = 0.28 | p = 0.28 |
|  | 0 v 6 | **p < 1^10-4**  **R^2^ = 0.10** | **p < 1^10-4**  **R^2^ = 0.25** | **p < 1^10^-4^**  **R^2^ = 0.07** | p = 0.06 |
| Clay | 0 v 3 | **p = 0.011**  **R^2^ = 0.07** | **p = 0.022**  **R^2^ = 0.12** | n.a. | n.a. |
|  | 3 v 6 | p = 0.54 | p = 0.08 | n.a. | n.a. |
|  | 0 v 6 | **p = 0.0004**  **R^2^ = 0.08** | **p = 0.0014**  **R^2^ = 0.15** | n.a. | n.a. |

**Table S8.** Differential abundance of bacterial mASVs between treatments prior to the emulated drought. The cells in red represent an increase in bacterial abundance with higher salinity, while the cells in blue represent a decrease in bacterial abundance with higher salinity. Only mASVs showing an adjusted p-value < 0.05 (Bonferroni correction) for both the ANCOMBC2 and the ALDEx2 methods are presented. mASVs relate to those detailed in the ‘16S_taxonomy.tsv’ and ‘16S_reqseqs.fasta’ files.

Clay

| **Method** | **ASV** | **Taxon** | 0dS/m-3dS/m | | 0dS/m-6dS/m | | 3dS/m-6dS/m | |  |
| --- | --- | --- | --- | --- | --- | --- | --- | --- | --- |
|  |  |  | Statistic | p-adj | Statistic | p-adj | Statistic | p-adj |  |
| ANCOMBC2 | eada5a254def2870f72b4f173e3ae333 | *Geobacillus* | 17.2 | 5.x10^-13^ | 21.4 | 3x10^-15^ | 2.298 | 1.000 |  |
| ALDEx2 | eada5a254def2870f72b4f173e3ae333 | *Geobacillus* | 11.0 | 0.0003 | 12.9 | 2x10^-5^ | 3.454 | 1.000 |  |

Sand

| **Method** | **ASV** | **Taxon** | 0dS/m-3dS/m | | 0dS/m-6dS/m | | 3dS/m-6dS/m | |  |
| --- | --- | --- | --- | --- | --- | --- | --- | --- | --- |
|  |  |  | Statistic | p-adj | Statistic | p-adj | Statistic | p-adj |  |
| ANCOMBC2 | 1bdf055f72d8e05f86ec7a9a0a775d11 | Rhizobiales | 2.562 | 1.000 | 4.825 | 0.004 | 2.066 | 1.000 |  |
|  | 7061db1184dc78b7dde4268090b2d3ff | Rhizobiaceae | 2.681 | 0.912 | 5.376 | 8x10^-4^ | 2.199 | 1.000 |  |
|  | bf30e92cee0af043ffbe4833dabbaabe | *Devosia* | 3.249 | 0.229 | 4.907 | 0.003 | 1.407 | 1.000 |  |
| ALDEx2 | 1bdf055f72d8e05f86ec7a9a0a775d11 | Rhizobiales | 3.228 | 1.000 | 5.925 | 0.045 | 3.813 | 0.967 |  |
|  | 7061db1184dc78b7dde4268090b2d3ff | Rhizobiaceae | 3.737 | 0.994 | 8.210 | 1x10^-4^ | 4.081 | 0.990 |  |
|  | bf30e92cee0af043ffbe4833dabbaabe | *Devosia* | 4.057 | 0.880 | 6.523 | 0.014 | 2.376 | 1.000 |  |

Silt – no differential abundance of bacterial mASVs identified.

**Table S9.** Differential abundance of fungal mASVs between treatments prior to the emulated drought. The cells in red represent an increase in fungal abundance with higher salinity, while the cells in blue represent a decrease in fungal abundance with higher salinity. Only mASVs showing an adjusted p-value < 0.05 (Bonferroni correction) for both the ANCOMBC2 and the ALDEx2 methods are presented. mASVs relate to those detailed in the ‘ITS_taxonomy.tsv’ and ‘ITS_reqseqs.fasta’ files.

Sand

| **Method** | **ASV** | **Taxon** | 0dS/m-3dS/m | | 0dS/m-6dS/m | | 3dS/m-6dS/m | |  |
| --- | --- | --- | --- | --- | --- | --- | --- | --- | --- |
|  |  |  | Statistic | p-adj | Statistic | p-adj | Statistic | p-adj |  |
| ANCOMBC2 | e8920db312239401749eaf638ee666b5 | Fungi | 1.995 | 1.00 | 4.80 | 0.021 | 2.02 | 1.000 |  |
|  | ebb63d7924977147ada4c808364b431b | Mortierellaceae | -1.508 | 1.00 | -5.16 | 0.008 | -3.19 | 1.000 |  |
| ALDEx2 | e8920db312239401749eaf638ee666b5 | Fungi | 2.985 | 0.99 | 6.41 | 0.016 | 3.56 | 1.000 |  |
|  | ebb63d7924977147ada4c808364b431b | Mortierellaceae | -1.622 | 1.00 | -6.65 | 0.005 | -4.70 | 0.915 |  |

Silt and clay– no differential abundance of fungal mASVs identified.

**Table S10.** Effects of irrigation treatment on abundance of key fungal diseases prior to the emulated drought. Analyses of min-max scaled normalized data by Kruskal-Wallis. Significant differences p < 0.05 are in bold. See Figure S2.

|  | Potatoes | | | Spinach | | |
| --- | --- | --- | --- | --- | --- | --- |
|  | *Alternaria thlaspis* | *Fusarium oxysporum* | *Fusarium venenatum* | *Alternaria thlaspis* | *Fusarium oxysporum* | *Fusarium venenatum* |
| Sand | Χ^2^ = 9.752  **p = 0.007** | Χ^2^ = 9.380  **p = 0.009** | Χ^2^ = 6.066  **p = 0.048** | Χ^2^ = 5.252  p = 0.072 | Χ^2^ = 0.620  p = 0.733 | Χ^2^ = 0.769  p = 0.681 |
| Silt | Χ^2^ = 2.000  p = 0.368 | Χ^2^ = 2.961  p = 0.228 | Χ^2^ = 2.356  p = 0.308 | Χ^2^ = 0.852  p = 0.653 | Χ^2^ = 1.215  p = 0.545 | Χ^2^ = 3.960  p = 0.138 |
| Clay | Χ^2^ = 0.960  p = 0.619 | Χ^2^ = 2.007  p = 0.367 | Χ^2^ = 2.500  p = 0.287 | Χ^2^ = 6.000  p = 0.050 | Χ^2^ = 1.470  p = 0.480 | n.a. |

**Table S11.** Effect of an emulated drought with 9 dS/m irrigation on numbers of bacterial and fungal mASVs (rarefied richness). P values from Kruskal-Wallis tests are tabled with eta^2^ effect sizes where p-values report significance <0.05 which are in bold.

|  | Sand | | Silt | | Clay | |
| --- | --- | --- | --- | --- | --- | --- |
| Pre-drought treatment | Bacteria | Fungi | Bacteria | Fungi | Bacteria | Fungi |
| 0 dS/m irrigation | **0.0059**  eta^2^ =0.41 | 0.79 | **0.0009**  eta^2^ = 0.68 | **0.013**  eta^2^ = 0.29 | **0.0015**  eta^2^ = 0.54 | 0.6 |
| 3 dS/m irrigation | **0.0009**  eta^2^ = 0.56 | 0.65 | **0.0025**  eta^2^ = 0.48 | **0.041**  eta^2^ = 0.18 | **0.0003**  eta^2^ = 0.68 | 0.33 |
| 6 dS/m irrigation | **0.035**  eta^2^ = 0.19 | 0.17 | 0.23 | 0.76 | **0.0002**  eta^2^ = 0.74 | 0.06 |

**Table S12**. Effect of an emulated drought with 9 dS/m irrigation on the types (presence/absence) of bacterial and fungal mASVs based on Jaccard dissimilarities. P values from PermANOVA tests (10,000 permutations) are tabled with R^2^ effect sizes as all p-values report significance <0.05. Percent effect size is 100 x R^2^.

**Table S13**. Effect of an emulated drought with 9 dS/m irrigation on the min-max scaled normalized abundances of bacterial and fungal mASVs based on Jaccard dissimilarities. P values from PermANOVA tests (10,000 permutations) are tabled with R^2^ effect sizes as all p-values report significance <0.05. Percent effect size is 100 x R^2^.

**Table S14.** Differential abundance of bacterial mASVs before and after 9dS/m irrigation between treatments. The cells in red represent an increase in bacterial abundance with higher salinity, while the cells in blue represent a decrease in bacterial abundance with higher salinity. Only mASVs showing an adjusted p-value < 0.05 (Bonferroni correction) for both the ANCOMBC2 and the ALDEx2 methods are presented. mASVs relate to those detailed in the ‘16S_taxonomy.tsv’ and ‘16S_reqseqs.fasta’ files.

Bacteria in silt for 0 dS/m irrigation

| **ASV** | **Taxon** | ANCOMBC2 | | | ALDEx2 | | |  |
| --- | --- | --- | --- | --- | --- | --- | --- | --- |
|  |  | Statistic | p-value | p-adj | Statistic | p-value | p-adj |  |
| 85ca5ad39abe485770c61f58964caf55 | Chitinophagaceae | 14.103 | 4.620e-10 | 2.473e-06 | 9.008 | 9.764e-06 | 0.048 |  |
| beaa91b73e829c507286c6e8e6c2a738 | Thermoactinomycetaceae | 22.489 | 5.731e-14 | 4.090e-09 | -10.465 | 2.488e-06 | 0.016 |  |
| 27d945a4964a2aeeab634c642180c259 | *Ilumatobacter* | 0.008 | 2.937e-06 | 0.003 | -7.174 | 5.040e-06 | 0.033 |  |

Bacteria in silt for 3dS/m irrigation

| **ASV** | **Taxon** | ANCOMBC2 | | | ALDEx2 | | |  |
| --- | --- | --- | --- | --- | --- | --- | --- | --- |
|  |  | Statistic | p-value | p-adj | Statistic | p-value | p-adj |  |
| 3cec2657123f75e4d09ba518c11bc53e | Thermomicrobiales | 11.779 | 1.337e-05 | 1.431e-05 | 8.303 | 9.608e-06 | 0.033 |  |
| 11035166eb57b1b63ec6ea1fba3a72ef | Lachnospiraceae | -5.596 | 3.211e-05 | 0.004 | -7.924 | 7.100e-07 | 0.004 |  |

Bacteria in clay for 0dS/m irrigation

| **ASV** | **Taxon** | ANCOMBC2 | | | ALDEx2 | | |  |
| --- | --- | --- | --- | --- | --- | --- | --- | --- |
|  |  | Statistic | p-value | p-adj | Statistic | p-value | p-adj |  |
| 3e9c671e2cb495635a8375dc67de9099 | Gammaproteobacteria | 4.911 | 1.320e-04 | 0.010 | 8.254 | 4.598e-07 | 0.002 |  |
| 8be86b58509424d594bb4d32c0e87091 | Chitinophagaceae | 5.257 | 6.420e-05 | 0.006 | 7.670 | 9.764e-07 | 0.005 |  |
| fd818098b320b16324938a447fddd04e | Burkholderiales | 4.832 | 1.561e-04 | 0.012 | 6.954 | 3.682e-06 | 0.018 |  |
| 295ed1b45986a9728f9d7f90ac67d401 | Burkholderiales | 4.258 | 5.310 | 0.033 | 7.309 | 6.353e-06 | 0.031 |  |
| 56bb681fb62a6803a743c65b45e7493c | *Flavitalea* | 9.321 | 4.298e-08 | 8.764e-06 | 7.499 | 1.469e-05 | 0.048 |  |
| 52b6fcb150595695ba31a2c3ff41218e | *Haliangium* | 4.437 | 3.612e-04 | 0.024 | 7.260 | 2.392e-06 | 0.012 |  |
| 92b9d6c24f01f5842106dc881b3adc15 | *Lysobacter* | 4.731 | 3.612e-04 | 0.024 | 6.806 | 3.612e-06 | 0.017 |  |
| 69cbc83a66d5a5db5dd0609c88c0162a | *Reyranella* | 27.519 | 1.541e-15 | 1.736e-12 | 11.673 | 6.563e-07 | 0.003 |  |
| 47b5aafdbfa6b4a89dccac6e8024e954 | *Steroidobacter* | 4.873 | 1.431e-04 | 0.011 | 7.605 | 1.626e-06 | 0.008 |  |
| 31c18e5a01cf19eab4958eab96967aae | Actinobacteriota | -18.151 | 1.452e-12 | 6.217e-10 | -8.375 | 3.285e-05 | 0.036 |  |
| 20db15662c1e2ef5df0cc0ba3b10f78d | Chloroflexi | -35.822 | 1.873e-17 | 5.014e-14 | -10.839 | 5.843e-06 | 0.011 |  |
| 64e19b3d1da44d12d3fc023108260b1b | Chloroflexi | -21.709 | 7.804e-14 | 5.063e-11 | -9.208 | 6.357e-06 | 0.022 |  |
| 32d1f2b3778220bffcbab0315e94df1b | Chloroflexi | -19.598 | 4.171e-13 | 2.126e-10 | -8.536 | 2.910e-06 | 0.014 |  |
| 5cb52abf8590417d80b8aa8abac009a6 | Chloroflexi | -18.188 | 1.404e-12 | 6.217e-10 | -8.938 | 4.767e-06 | 0.023 |  |
| e05e972fcf21db0dd2a5bb6c5a74c0d1 | Chloroflexi | -14.934 | 3.321e-11 | 1.147e-08 | -7.713 | 9.827e-06 | 0.043 |  |
| ebf9c7c66db43f6e2baab89cfdf12d41 | Chloroflexi | -14.510 | 5.241e-11 | 1.726e-08 | -8.026 | 5.776e-06 | 0.028 |  |
| 4bdb5af38bf69bad9f9fbbce2634e020 | Holophagae | -21.324 | 1.047e-13 | 6.404e-11 | -8.706 | 7.353e-06 | 0.035 |  |
| 6c6492ce2bad8705d41061ee5062a1eb | Chthoniobacterales | -18.519 | 1.048e-12 | 4.776e-10 | -8.672 | 3.130e-06 | 0.015 |  |
| 642d2cb47bb7b74eb4ecaf2520460905 | Thermomicrobiales | -55.615 | 1.126e-20 | 1.206e-16 | -9.455 | 4.790e-06 | 0.022 |  |
| fbeeae7a656ec50dfd0a017d4e68b44e | Thermomicrobiales | -36.691 | 1.252e-17 | 3.830e-14 | -9.475 | 2.454e-05 | 0.032 |  |
| aa548b25c86c045cab32a0b24b2473ab | Thermomicrobiales | -32.818 | 8.137e-17 | 1.340e-13 | -9.886 | 3.480e-06 | 0.017 |  |
| 423b462d87749ef98eee20b8a58a710f | Thermomicrobiales | -22.992 | 3.032e-14 | 2.094e-11 | -8.530 | 5.396e-06 | 0.026 |  |
| e8c311d8e25777f77b63be45d5191fd0 | Thermomicrobiales | -17.921 | 1.784e-12 | 7.207e-10 | -7.892 | 6.366e-06 | 0.030 |  |
| 21f1e8d9461147b629e97932631f3bf7 | Vicinamibacterales | -37.164 | 1.010e-17 | 3.830e-14 | -10.766 | 1.556e-06 | 0.007 |  |
| c5a69722637cf33f1c6f3c2cec39ef7e | Vicinamibacterales | -19.998 | 2.998e-13 | 1.566e-10 | -8.169 | 6.224e-06 | 0.030 |  |
| 56409560f384177a2cb5af33b6931c6f | Vicinamibacteraceae | -25.331 | 6.103e-15 | 5.226e-12 | -9.427 | 2.252e-05 | 0.024 |  |
| 6bec10ce7f7c55d25772e394e70747cc | *Microlunatus* | -54.058 | 1.821e-20 | 1.299e-16 | -12.341 | 8.407e-06 | 0.008 |  |
| e717e93334705a44aefed099f267880a | *Paenibacillus* | -57.249 | 6.902e-21 | 1.206e-16 | -8.440 | 9.693e-06 | 0.040 |  |
| 4b8eea3e07f9e9ac389efb29bdbbe897 | *Rhodobaculum* | -33.365 | 6.170e-17 | 1.101e-13 | -9.695 | 9.735e-06 | 0.023 |  |
| ff0bd87fd9651325f14f76be6e315023 | *Udaeobacter* | -24.597 | 9.935e-15 | 7.842e-12 | -8.636 | 5.331e-06 | 0.025 |  |
| 698e9071fa33454284e6b4ee8fec8eba | *Udaeobacter* | -20.933 | 1.419e-13 | 7.996e-11 | -9.492 | 2.039e-06 | 0.010 |  |

Bacteria in clay for 3dS/m irrigation

| **ASV** | **Taxon** | ANCOMBC2 | | | ALDEx2 | |  |  |  |
| --- | --- | --- | --- | --- | --- | --- | --- | --- | --- |
|  |  | Statistic | p-value | p-adj | Statistic | p-value | | p-adj |  |
| 2960e54f7b74ae925ef4f209255a16ab | Gemmatimonadaceae | 30.527 | 5.892e-17 | 6.308e-14 | 9.539 | 6.989e-06 | | 0.036 |  |
| e78b5a4ac7083d74ce359349f5be62a1 | Ilumatobacteraceae | 37.634 | 1.440e-18 | 2.569e-15 | 9.721 | 5.971e-06 | | 0.024 |  |
| 7ad4351125dedf3b029ac95b8daf62ed | *Amaricoccus* | 41.416 | 2.617e-19 | 6.031e-16 | 11.759 | 2.245e-07 | | 0.001 |  |
| 1ffa1df945126284fd248c0e4851d2f9 | *Acidibacter* | 6.150 | 8.308e-06 | 9.694e-04 | 9.309 | 3.558e-07 | | 0.002 |  |
| 69cbc83a66d5a5db5dd0609c88c0162a | *Reyranella* | 32.940 | 1.533e-17 | 1.931e-14 | 11.727 | 5.387e-07 | | 0.003 |  |
| 31c18e5a01cf19eab4958eab96967aae | Actinobacteriota | -33.669 | 1.040e-17 | 1.485e-14 | -10.578 | 2.990e-06 | | 0.014 |  |
| 5d5a4e02f8c59855251e335dc882acc6 | Chloroflexi | -41.026 | 3.099e-19 | 6.031e-16 | -9.100 | 1.506e-05 | | 0.038 |  |
| 5cb52abf8590417d80b8aa8abac009a6 | Chloroflexi | -19.044 | 2.240e-13 | 9.993e-11 | -9.036 | 2.097e-06 | | 0.010 |  |
| 4bdb5af38bf69bad9f9fbbce2634e020 | Holophagae | -18.305 | 4.417e-13 | 1.854e-10 | -8.065 | 1.098e-05 | | 0.043 |  |
| 87a6eefa6e996c377b6b5447504720ad | Thermoleophilia | -53.111 | 3.082e-21 | 2.199e-17 | -8.738 | 1.836e-05 | | 0.048 |  |
| 6c6492ce2bad8705d41061ee5062a1eb | Chthoniobacterales | -16.562 | 2.430e-12 | 9.291e-10 | -8.812 | 1.787e-06 | | 0.009 |  |
| 26205cee38640d934a0df582e36bad22 | Rokubacteriales | -24.296 | 3.269e-15 | 2.059e-12 | -8.460 | 9.802e-06 | | 0.045 |  |
| 09cce70cf15e90cacb1c60f1bb790794 | Rokubacteriales | -29.464 | 1.102e-16 | 1.026e-13 | -7.990 | 1.039e-05 | | 0.047 |  |
| 642d2cb47bb7b74eb4ecaf2520460905 | Thermomicrobiales | -49.711 | 1.006e-27 | 4.309e-17 | -10.412 | 4.28e-06 | | 0.013 |  |
| 1f085927e5e47e35de3c592dd6515ea5 | Thermomicrobiales | -46.010 | 4.008e-20 | 1.073e-16 | -8.944 | 1.708e-05 | | 0.049 |  |
| f7f63df19b681f488d289898f4fea4aa | Thermomicrobiales | -41.136 | 2.954e-19 | 6.031e-16 | -10.585 | 7.610e-07 | | 0.004 |  |
| d4d0792162076a27259e794fcd4373dd | Thermomicrobiales | -30.916 | 4.712e-17 | 5.310e-14 | -8.461 | 4.098e-06 | | 0.020 |  |
| 423b462d87749ef98eee20b8a58a710f | Thermomicrobiales | -30.191 | 7.171e-17 | 7.311e-14 | -9.505 | 9.555e-06 | | 0.022 |  |
| aa548b25c86c045cab32a0b24b2473ab | Thermomicrobiales | -28.215 | 2.367e-16 | 2.027e-13 | -9.596 | 5.129e-06 | | 0.019 |  |
| e8c311d8e25777f77b63be45d5191fd0 | Thermomicrobiales | -23.417 | 6.225e-15 | 3.808e-12 | -9.204 | 1.157e-05 | | 0.023 |  |
| fbeeae7a656ec50dfd0a017d4e68b44e | Thermomicrobiales | -18.764 | 2.890e-13 | 1.262e-10 | -8.158 | 7.950e-06 | | 0.029 |  |
| 914070a5921d7712507bbbfadba340ca | Vicinamibacterales | -36.870 | 2.074e-18 | 3.416e-15 | -9.065 | 8.410e-06 | | 0.032 |  |
| 21f1e8d9461147b629e97932631f3bf7 | Vicinamibacterales | -33.340 | 1.238e-17 | 1.657e-14 | -11.293 | 5.271e-07 | | 0.003 |  |
| 8d4e3d9590b5d11b757cc918c4da0592 | Vicinamibacterales | -19.871 | 1.077e-13 | 5.242e-11 | -8.274 | 7.243e-06 | | 0.033 |  |
| 8db57df79fb75fa3c6829a966f58928d | Vicinamibacterales | -17.834 | 6.902e-13 | 2.736e-10 | -7.826 | 7.931e-06 | | 0.039 |  |
| 56409560f384177a2cb5af33b6931c6f | Vicinamibacteraceae | -25.859 | 1.097e-15 | 8.099e-13 | -9.333 | 4.726e-06 | | 0.022 |  |
| 7b5ce8096f0b5e91be6f0917a328c92f | Vicinamibacteraceae | -22.004 | 1.842e-14 | 1.011e-11 | -8.170 | 1.472e-05 | | 0.042 |  |
| 1d1fe3380e5ac53d8a062f10f5f4841f | Vicinamibacteraceae | -20.897 | 4.511e-14 | 2.355e-11 | -9.111 | 3.466e-06 | | 0.017 |  |
| a3cab5660a856b609232b6e984a3def5 | *Bacillus* | -48.131 | 1.792e-20 | 5.831e-17 | -9.331 | 9.385e-06 | | 0.039 |  |
| 6bec10ce7f7c55d25772e394e70747cc | *Microlunatus* | -70.251 | 2.053e-23 | 3.702e-19 | -12.203 | 5.126e-08 | | 2.533e-04 |  |
| 4b8eea3e07f9e9ac389efb29bdbbe897 | *Rhodobaculum* | -68.239 | 2.053e-23 | 3.702e-19 | -10.704 | 1.543e-05 | | 0.012 |  |
| 698e9071fa33454284e6b4ee8fec8eba | *Udaeobacter* | -18.356 | 4.213e-13 | 1.804e-10 | -10.003 | 4.040e-07 | | 0.002 |  |
| ff0bd87fd9651325f14f76be6e315023 | *Udaeobacter* | -15.980 | 4.451e-12 | 1.615e-09 | -8.595 | 6.122e-06 | | 0.022 |  |
| 566a409979185fb9cdd650520863823c | *Udaeobacter* | -15.446 | 7.890e-12 | 2.769e-09 | -8.931 | 1.599e-05 | | 0.018 |  |
| 06b8eed98b034c36998df719045cf7b6 | *Udaeobacter* | -12.015 | 4.947e-10 | 1.558e-07 | -7.399 | 5.612e-06 | | 0.027 |  |
| 425dbf5cc6cbced5af9821fffc06e6ba | *Udaeobacter* | -11.878 | 5.946e-10 | 1.836e-07 | -7.799 | 9.622e-06 | | 0.041 |  |
| 82ebd345f729aa39b7b13860fcb658ad | *Udaeobacter* | -11.733 | 7.241e-10 | 2.153e-07 | -7.983 | 3.311e-06 | | 0.016 |  |
| 4f183f8a070b55ba17f7c0d81b368c70 | *Udaeobacter* | -11.170 | 1.585e-09 | 4.586e-07 | -7.129 | 6.940e-06 | | 0.034 |  |

Bacteria in clay for 6dS/m irrigation

| **ASV** | **Taxon** | ANCOMBC2 | | | ALDEx2 | | |  |
| --- | --- | --- | --- | --- | --- | --- | --- | --- |
|  |  | Statistic | p-value | p-adj | Statistic | p-value | p-adj |  |
| 3e9c671e2cb495635a8375dc67de9099 | Gammaproteobacteria | 7.059 | 1.388e-06 | 1.955e-04 | 9.266 | 6.963e-08 | 3.732e-04 |  |
| 83a02254db1a07f262f437f24013f34f | Acidobacteriales | 7.388 | 7.465e-07 | 1.097e-04 | 8.145 | 1.023e-06 | 0.005 |  |
| fd818098b320b16324938a447fddd04e | Burkholderiales | 5.070 | 7.974e-05 | 0.007 | 6.433 | 8.416e-06 | 0.045 |  |
| aec82c1f152e9a10ad64f1501b651031 | Burkholderiales | 4.790 | 1.465e-04 | 0.012 | 7.510 | 9.406e-07 | 0.005 |  |
| c6ea8fdb2562bae0809fba13a4d17809 | Comamonadaceae | 4.737 | 1.645e-04 | 0.013 | 6.756 | 3.461e-06 | 0.019 |  |
| e20765757b77a74212c897a970eab2f4 | Microscillaceae | 15.503 | 7.416e-12 | 2.175e-09 | 9.347 | 3.113e-06 | 0.014 |  |
| 1cd5803fc14d25df9b37f01a82e4623a | Myxococcaceae | 6.329 | 5.781e-06 | 7.281e-04 | 7.969 | 6.839e-07 | 0.004 |  |
| 9671146a3a948da12762a8dba6467fcc | Sphingomonadaceae | 5.022 | 8.851e-05 | 0.008 | 7.036 | 1.525e-06 | 0.008 |  |
| ba64834f318ae70416a47379d0a1e796 | Steroidobacteraceae | 5.169 | 6.448e-05 | 0.006 | 6.660 | 4.322e-06 | 0.023 |  |
| 8dea7fc3ac6bfbd91da77ce0b8d8b2e4 | *Antarcticibacterium* | 9.521 | 1.891e-08 | 3.892e-06 | 7.435 | 3.541e-06 | 0.019 |  |
| 43fb9aa788ed2678376927a64a44e85b | *Bryobacter* | 5.608 | 2.540e-05 | 0.002 | 7.538 | 1.062e-06 | 0.006 |  |
| 2c88957fdba829fb10b396b49ea22b27 | *Gramella* | 11.022 | 1.958e-09 | 4.366e-07 | 8.167 | 1.614e-06 | 0.009 |  |
| 92b9d6c24f01f5842106dc881b3adc15 | *Lysobacter* | 4.821 | 1.369e-04 | 0.011 | 6.283 | 7.396e-06 | 0.040 |  |
| acd051230390d6cdc9c08e32b01adc9b | *Nitrospira* | 5.170 | 6.437e-05 | 0.006 | 7.029 | 2.597e-06 | 0.014 |  |
| 69cbc83a66d5a5db5dd0609c88c0162a | *Reyranella* | 41.413 | 2.621e-19 | 2.551e-16 | 11.946 | 1.311e-06 | 0.007 |  |
| 47b5aafdbfa6b4a89dccac6e8024e954 | *Steroidobacter* | 6.143 | 8.413e-06 | 9.843e-04 | 8.531 | 3.791e-07 | 0.002 |  |
| 31c18e5a01cf19eab4958eab96967aae | Actinobacteriota | -72.912 | 1.054e-23 | 2.821e-20 | -11.508 | 7.837e-07 | 0.004 |  |
| 20db15662c1e2ef5df0cc0ba3b10f78d | Chloroflexi | -108.375 | 8.537e-27 | 9.139e-23 | -12.401 | 2.390e-07 | 0.001 |  |
| 64e19b3d1da44d12d3fc023108260b1b | Chloroflexi | -85.706 | 5.787e-25 | 3.098e-21 | -11.059 | 1.278e-06 | 0.007 |  |
| 687b93830995221fa41cadb3e60efaff | Chloroflexi | -56.529 | 1.009e-21 | 2.161e-18 | -9.678 | 2.057e-06 | 0.011 |  |
| 5cb52abf8590417d80b8aa8abac009a6 | Chloroflexi | -50.691 | 7.097e-21 | 1.169e-17 | -10.583 | 5.250e-06 | 0.010 |  |
| 916eec2366e22250d805c152ada19a1f | Chloroflexi | -47.565 | 2.214e-20 | 3.159e-17 | -8.683 | 4.186e-06 | 0.022 |  |
| 5d5a4e02f8c59855251e335dc882acc6 | Chloroflexi | -44.164 | 8.325e-20 | 9.381e-17 | -9.734 | 4.594e-06 | 0.014 |  |
| 4993814b7bafe8993326c418417d6529 | Chloroflexi | -37.299 | 1.689e-18 | 1.506e-15 | -8.485 | 2.627e-05 | 0.048 |  |
| 4d780939144d85909613a3586656903d | Chloroflexi | -17.080 | 1.440e-12 | 4.469e-10 | -8.988 | 3.622e-06 | 0.018 |  |
| e05e972fcf21db0dd2a5bb6c5a74c0d1 | Chloroflexi | -15.360 | 8.666e-12 | 2.441e-09 | -8.497 | 3.193e-06 | 0.015 |  |
| ebf9c7c66db43f6e2baab89cfdf12d41 | Chloroflexi | -14.590 | 2.047e-11 | 5.619e-09 | -7.985 | 1.737e-05 | 0.033 |  |
| fe71798780184c48d6bd2e0553cd3363 | Holophagae | -49.839 | 9.610e-21 | 1.470e-17 | -8.885 | 2.589e-05 | 0.036 |  |
| 6c6492ce2bad8705d41061ee5062a1eb | Chthoniobacterales | -26.775 | 5.950e-16 | 3.033e-13 | -11.353 | 6.739e-07 | 0.004 |  |
| 87a6eefa6e996c377b6b5447504720ad | Thermoleophilia | -53.063 | 3.132e-21 | 6.067e-18 | -9.267 | 8.934e-06 | 0.029 |  |
| f7f63df19b681f488d289898f4fea4aa | Thermomicrobiales | -117.135 | 2.111e27 | 4.520e-23 | -11.749 | 1.137e-06 | 0.006 |  |
| e8c311d8e25777f77b63be45d5191fd0 | Thermomicrobiales | -98.518 | 4.738e-26 | 3.381e-22 | -11.618 | 4.042e-06 | 0.021 |  |
| 423b462d87749ef98eee20b8a58a710f | Thermomicrobiales | -73.397 | 9.356e-24 | 2.821e-20 | -9.643 | 1.828e-06 | 0.010 |  |
| 642d2cb47bb7b74eb4ecaf2520460905 | Thermomicrobiales | -52.819 | 3.401e-21 | 6.067e-18 | -10.605 | 6.593e-06 | 0.020 |  |
| fbeeae7a656ec50dfd0a017d4e68b44e | Thermomicrobiales | -43.914 | 9.212e-20 | 9.861e-17 | -10.610 | 7.975e-07 | 0.004 |  |
| 53378cd1e8a390dc1c587e03d2613f24 | Thermomicrobiales | -30.887 | 4.792e-17 | 3.309e-14 | -9.300 | 2.194e-06 | 0.012 |  |
| d4d0792162076a27259e794fcd4373dd | Thermomicrobiales | -30.049 | 7.788e-17 | 5.211e-14 | -8.831 | 2.624e-06 | 0.014 |  |
| aa548b25c86c045cab32a0b24b2473ab | Thermomicrobiales | -25.019 | 1.957e-15 | 9.110e-13 | -9.261 | 4.118e-06 | 0.022 |  |
| 26205cee38640d934a0df582e36bad22 | Rokubacteriales | -45.435 | 5.017e-20 | 5.968e-17 | -9.626 | 1.681e-06 | 0.009 |  |
| 6233ed6152f974791ea89c8013b2f779 | Rokubacteriales | -39.283 | 6.713e-19 | 6.249e-16 | -8.360 | 8.670e-06 | 0.038 |  |
| 6f125e1d73398f7407ee2d19c8082557 | Vicinamibacterales | -42.186 | 1.885e-19 | 1.922e-16 | -9.426 | 1.753e-06 | 0.009 |  |
| c5a69722637cf33f1c6f3c2cec39ef7e | Vicinamibacterales | -25.328 | 1.578e-15 | 7.506e-13 | -8.893 | 1.728e-05 | 0.044 |  |
| db6be6082965a96abbbc06b115af2d09 | Vicinamibacterales | -14.325 | 2.777e-11 | 7341e-09 | -7.393 | 1.922e-05 | 0.047 |  |
| 21f1e8d9461147b629e97932631f3bf7 | Vicinamibacterales | -13.852 | 4.847e-11 | 1.221e-08 | -7.666 | 5.017e-06 | 0.026 |  |
| 328132e46d8f5c7244e1d85a7f777455 | Roseiflexaceae | -31.001 | 4.489e-17 | 3.204e-14 | -10.760 | 2.334e-06 | 0.010 |  |
| 1d1fe3380e5ac53d8a062f10f5f4841f | Vicinamibacteraceae | -28.263 | 2.297e-16 | 1.405e-13 | -9.485 | 6.495e-06 | 0.029 |  |
| 5fdd2194166ecdb1e6ba3746b03ceb4a | *Hydrogenispora* | -33.883 | 9.296e-18 | 7.961e-15 | -8.644 | 6.058e-06 | 0.032 |  |
| 9abc193ef8aac6b075fafeccbafef8a5 | *Luedemannella* | -84.304 | 7.782e-25 | 3.332e-21 | -9.734 | 9.662e-06 | 0.025 |  |
| 6bec10ce7f7c55d25772e394e70747cc | *Microlunatus* | -61.961 | 1.951e-22 | 4.641e-19 | -12.449 | 1.008e-07 | 5.353e-04 |  |
| 4b8eea3e07f9e9ac389efb29bdbbe897 | *Rhodobaculum* | -47.358 | 2.393e-20 | 3.202e-17 | -11.002 | 3.887e-07 | 0.002 |  |
| 54562c2e014c13532991bdf0cbd446f6 | *Thermostaphylospora* | -14.104 | 3.597e-11 | 9.391e-09 | -7.773 | 1.379e-06 | 0.030 |  |
| 698e9071fa33454284e6b4ee8fec8eba | *Udaeobacter* | -32.400 | 2.054e-17 | 1.692e-14 | -11.670 | 1.140e-07 | 6.041e-04 |  |
| 7538c37bcb2f492b4a7c89174e7873f1 | *Udaeobacter* | -29.076 | 1.393e-16 | 8.770e-14 | -8.427 | 3.232e-05 | 0.044 |  |
| 82ebd345f729aa39b7b13860fcb658ad | *Udaeobacter* | -28.179 | 2.420e-16 | 1.439e-13 | -10.127 | 7.597e-07 | 0.004 |  |
| 425dbf5cc6cbced5af9821fffc06e6ba | *Udaeobacter* | -27.659 | 3.359e-16 | 1.844e-13 | -9.612 | 1.884e-06 | 0.010 |  |
| ff0bd87fd9651325f14f76be6e315023 | *Udaeobacter* | -22.106 | 1.699e-14 | 6.994e-12 | -9.580 | 4.249e-06 | 0.022 |  |
| 1c33e223f1007a11291157606e9bf305 | *Udaeobacter* | -20.743 | 5.128e-14 | 1.961e-11 | -8.766 | 1.021e-05 | 0.033 |  |
| 31941f9af31d93fc15c8bb19244d141f | *Udaeobacter* | -19.085 | 3.212e-31 | 7.107e-11 | -8.539 | 8.881e-06 | 0.029 |  |
| a3547724e7b579e332cf1c5b61c85901 | *Udaeobacter* | -18.754 | 2.914e-13 | 9.453e-14 | -7.953 | 8.845e-06 | 0.043 |  |
| 566a409979185fb9cdd650520863823c | *Udaeobacter* | -15.382 | 8.459e-12 | 2.415e-09 | -7.684 | 1.826e-05 | 0.043 |  |
| 06b8eed98b034c36998df719045cf7b6 | *Udaeobacter* | -13.460 | 7.783e-11 | 1.938e-08 | -7.631 | 6.364e-06 | 0.022 |  |
| 4f183f8a070b55ba17f7c0d81b368c70 | *Udaeobacter* | -11.521 | 9.694e-10 | 2.232e-07 | -7.172 | 1.101e-05 | 0.035 |  |

Bacteria in sand for 0dS/m irrigation

| **ASV** | **Taxon** | ANCOMBC2 | | | ALDEx2 | | |  |
| --- | --- | --- | --- | --- | --- | --- | --- | --- |
|  |  | Statistic | p-value | p-adj | Statistic | p-value | p-adj |  |
| fb803bdfbd439d4df2bca33b6b706524 | Bacteria | 5.236 | 8.149e-05 | 0.008 | 7.996 | 9.652e-07 | 0.005 |  |
| 4afcd38debcb1ee03f58bef8a42b31fe | Gammaproteobacteria | 6.873 | 3.750e-06 | 5.614e-04 | 9.108 | 5.736e-07 | 0.003 |  |
| 3e9c671e2cb495635a8375dc67de9099 | Gammaproteobacteria | 5.602 | 3.975e-05 | 0.004 | 7.541 | 3.146e-06 | 0.016 |  |
| 0476aeb17adab42fbeb06d8a396599e4 | Burkholderiales | 8.337 | 3.235e-07 | 6.075e-05 | 11.424 | 1.078e-08 | 5.656e-05 |  |
| aec82c1f152e9a10ad64f1501b651031 | Burkholderiales | 5.199 | 8.782e-05 | 0.008 | 6.993 | 4.365e-06 | 0.023 |  |
| 0648ca946cc84b68b053c7d8ddd059d9 | Rhizobiales | 4.262 | 5.962e-04 | 0.043 | 7.223 | 4.774e-06 | 0.025 |  |
| c6d99285210d97b4efabd735eb85e3ab | Rhizobiales | 4.665 | 2.590e-04 | 0.021 | 7.126 | 6.833e-06 | 0.036 |  |
| 60a011c012e89b3d260e2a6695edf7f5 | Caulobacteraceae | 6.387 | 9.004e-06 | 0.001 | 10.582 | 2.5841e-08 | 1.355e-04 |  |
| 7061db1184dc78b7dde4268090b2d3ff | Rhizobiaceae | 5.812 | 2.650e-05 | 0.003 | 10.248 | 3.562e-08 | 1.868e-04 |  |
| ba64834f318ae70416a47379d0a1e796 | Steroidobacteraceae | 7.356 | 1.620e-06 | 2.588e-04 | 9.514 | 1.322e-07 | 6.926e-04 |  |
| 1ffa1df945126284fd248c0e4851d2f9 | *Acidibacter* | 6.084 | 1.581e-05 | 0.002 | 7.817 | 1.722e-06 | 0.009 |  |
| 7ad4351125dedf3b029ac95b8daf62ed | *Amaricoccus* | 12.435 | 1.226e-09 | 3.645e-07 | 9.034 | 3.489e-06 | 0.018 |  |
| 4595f7b08c508155a49f8be016393016 | *Bauldia* | 6.371 | 9.274e-06 | 0.001 | 7.993 | 2.912e-06 | 0.015 |  |
| bf30e92cee0af043ffbe4833dabbaabe | *Devosia* | 5.893 | 2.270e-05 | 0.003 | 8.583 | 5.818e-07 | 0.003 |  |
| acd051230390d6cdc9c08e32b01adc9b | *Nitrospira* | 6.193 | 1.289e-05 | 0.002 | 10.971 | 3.771e-08 | 1.977e-04 |  |
| b50c4d0eaf89c73bbb27f54862757651 | *Rhodoplanes* | 5.415 | 5.721e-05 | 0.006 | 9.644 | 7.608e-08 | 3.987e-04 |  |
| 92b9d6c24f01f5842106dc881b3adc15 | *Lysobacter* | 5.481 | 5.031e-05 | 0.005 | 7.456 | 1.943e-06 | 0.010 |  |
| 31c18e5a01cf19eab4958eab96967aae | Actinobacteriota | -37.419 | 5.239e-17 | 1.119e-13 | -9.330 | 7.284e-05 | 0.045 |  |
| 20db15662c1e2ef5df0cc0ba3b10f78d | Chloroflexi | -37.218 | 5.705e-17 | 1.119e-13 | -11.819 | 4.288e-07 | 0.002 |  |
| 64e19b3d1da44d12d3fc023108260b1b | Chloroflexi | -37.048 | 6.133e-17 | 1.119e-13 | -10.827 | 3.596e-06 | 0.019 |  |
| af1bc90ea549e2ac70f4548e96fba5b4 | Chloroflexi | -25.787 | 1.843e-14 | 1.361e-11 | -9.596 | 1.648e-05 | 0.031 |  |
| 5cb52abf8590417d80b8aa8abac009a6 | Chloroflexi | -18.564 | 3.009e-12 | 1.371e-09 | -9.310 | 5.149e-06 | 0.026 |  |
| e05e972fcf21db0dd2a5bb6c5a74c0d1 | Chloroflexi | -12.506 | 1.129e-09 | 3.452e-07 | -7.883 | 7.967e-06 | 0.041 |  |
| de0067043d9f036b4b226b9124157d78 | Dehalococcoidia | -22.741 | 1.310e-13 | 7.579e-11 | -9.182 | 3.802e-06 | 0.020 |  |
| 87a6eefa6e996c377b6b5447504720ad | Thermoleophilia | -59.998 | 2.893e-20 | 3.311e-16 | -10.652 | 3.131e-06 | 0.016 |  |
| 60d75dfe767bf6e0b3ace47dd76e4459 | Thermomicrobiales | -36.415 | 8.057e-17 | 1.327e-13 | -11.731 | 5.564e-07 | 0.003 |  |
| f7f63df19b681f488d289898f4fea4aa | Thermomicrobiales | -37.315 | 5.476e-17 | 1.119e-13 | -9.411 | 1.625e-05 | 0.049 |  |
| d2273237c6a9e43ce23119beba3bbf1f | Thermomicrobiales | -30.989 | 1.029e-15 | 1.224e-12 | -9.273 | 1.185e-05 | 0.035 |  |
| 423b462d87749ef98eee20b8a58a710f | Thermomicrobiales | -16.165 | 2.477e-11 | 9.642e-09 | -8.456 | 3.517e-06 | 0.018 |  |
| e8c311d8e25777f77b63be45d5191fd0 | Thermomicrobiales | -12.526 | 1.103e-09 | 3.421e-07 | -8.228 | 1.088e-05 | 0.022 |  |
| 78874c5be28095183acb705c6683a40c | Vicinamibacterales | -38.387 | 3.497e-17 | 1.069e-13 | -10.610 | 2.906e-06 | 0.015 |  |
| 94f168bf1d4758457d5751b8e97a267d | Vicinamibacterales | -42.280 | 7.562e-18 | 4.048e-14 | -10.528 | 4.520e-06 | 0.019 |  |
| 823b95422fffd15930389bd1200b42e4 | Vicinamibacterales | -27.611 | 6.317e-15 | 6.148e-12 | -9.709 | 5.918e-06 | 0.026 |  |
| 914070a5921d7712507bbbfadba340ca | Vicinamibacterales | -29.371 | 2.393e-15 | 2.697e-12 | -9.554 | 4.413e-06 | 0.023 |  |
| db6be6082965a96abbbc06b115af2d09 | Vicinamibacterales | -22.974 | 1.118e-13 | 6.647e-11 | -9.497 | 1.015e-05 | 0.030 |  |
| 1d1fe3380e5ac53d8a062f10f5f4841f | Vicinamibacteraceae | -25.152 | 2.723e-14 | 1.943e-11 | -10.101 | 2.214e-05 | 0.035 |  |
| a3cab5660a856b609232b6e984a3def5 | *Bacillus* | -24.847 | 3.294e-14 | 2.137e-11 | -9.114 | 3.724e-06 | 0.019 |  |
| 6bec10ce7f7c55d25772e394e70747cc | *Microlunatus* | -59.747 | 3.093e-20 | 3.311e-16 | -13.511 | 1.076e-06 | 0.006 |  |
| 4b8eea3e07f9e9ac389efb29bdbbe897 | *Rhodobaculum* | -33.393 | 3.169e-16 | 4.523e-13 | -11.090 | 3.456e-06 | 0.011 |  |
| 4f183f8a070b55ba17f7c0d81b368c70 | *Udaeobacter* | -20.347 | 7.336e-13 | 3.654e-10 | -8.289 | 1.482e-05 | 0.045 |  |

Bacteria in sand for 3dS/m irrigation

| **ASV** | **Taxon** | ANCOMBC2 | | | ALDEx2 | | |  |
| --- | --- | --- | --- | --- | --- | --- | --- | --- |
|  |  | Statistic | p-value | p-adj | Statistic | p-value | p-adj |  |
| 0476aeb17adab42fbeb06d8a396599e4 | Burkholderiales | 6.281 | 6.368e-06 | 7.214e-04 | 9.893 | 3.048e-08 | 1.554e-04 |  |
| 93eeadb6d76f9980da7db506d202681b | Micrococcales | 4.826 | 1.354e-04 | 0.010 | 7.292 | 1.782e-06 | 0.009 |  |
| c6ea8fdb2562bae0809fba13a4d17809 | Comamonadaceae | 7.906 | 2.899e-07 | 4.371e-05 | 11.740 | 2.850e-09 | 1.454e-05 |  |
| 9f209d3a93f8cd248b0dd28bd4a4451a | Gemmatimonadaceae | 4.445 | 3.125e-04 | 0.019 | 6.782 | 5.940e-06 | 0.030 |  |
| 24f3cb6d61d69875b0ee108c1d263e68 | Ilumatobacteraceae | 6.985 | 1.597e-06 | 2.073e-04 | 12.145 | 7.545e-10 | 3.850e-06 |  |
| 7061db1184dc78b7dde4268090b2d3ff | Rhizobiaceae | 3.989 | 8.604e-04 | 0.046 | 7.917 | 4.039e-07 | 0.002 |  |
| 2ee9f2a345b4a6d195155de3973c7aca | Sandaracinaceae | 9.815 | 1.190e-08 | 2.548e-06 | 10.637 | 2.418e-08 | 1.232e-04 |  |
| 7ad4351125dedf3b029ac95b8daf62ed | *Amaricoccus* | 28.400 | 2.109e-16 | 1.806e-13 | 12.145 | 2.939e-07 | 0.001 |  |
| 57a988dcf233ad72d2749762cf1dc2fc | *Amaricoccus* | 31.151 | 4.120e-17 | 4.200e-14 | 10.117 | 2.897e-06 | 0.013 |  |
| 4595f7b08c508155a49f8be016393016 | *Bauldia* | 4.472 | 2.949e-04 | 0.018 | 7.764 | 2.506e-06 | 0.013 |  |
| 43fb9aa788ed2678376927a64a44e85b | *Bryobacter* | 4.939 | 1.061e-04 | 0.008 | 8.014 | 3.410e-07 | 0.002 |  |
| 02c0389a0c1f7b291e7f2e4538920676 | *Clostridium* | 56.520 | 1.012e-21 | 4.334e-18 | 10.266 | 1.442e-06 | 0.007 |  |
| 86fe369992720b5115fcb0e9426f9e8c | *Gemmatimonas* | 22.732 | 1.045e-14 | 5.735e-12 | 9.515 | 1.128e-05 | 0.031 |  |
| 25201dd814c297e17df2e1487ceb81b2 | *Gemmatimonas* | 6.074 | 9.687e-06 | 9.558e-04 | 7.672 | 7.558e-07 | 0.004 |  |
| 631ce11b19b2700a5126e21fccb69e2e | *Gemmatimonas* | 5.168 | 6.458e-05 | 0.005 | 7.037 | 9.032e-06 | 0.046 |  |
| 92b9d6c24f01f5842106dc881b3adc15 | *Lysobacter* | 5.301 | 4.866e-05 | 0.004 | 7.831 | 6.153e-07 | 0.003 |  |
| cda56b595263196a226721cdf89d626e | *Microvirga* | 4.472 | 2.947e-04 | 0.018 | 8.320 | 2.350e-07 | 0.001 |  |
| 8352c77bebdd5805daa430aabfd1d786 | *Nitrospira* | 5.389 | 4.029e-05 | 0.003 | 7.140 | 6.700e-06 | 0.034 |  |
| 47b5aafdbfa6b4a89dccac6e8024e954 | Steroidobacter | 6.521 | 3.944e-06 | 4.798e-04 | 8.484 | 2.220e-07 | 0.001 |  |
| 31c18e5a01cf19eab4958eab96967aae | Actinobacteriota | -41.236 | 2.829e-19 | 5.505e-16 | -9.077 | 3.399e-06 | 0.017 |  |
| e05e972fcf21db0dd2a5bb6c5a74c0d1 | Chloroflexi | -52.771 | 3.456e-21 | 1.233e-17 | -11.495 | 1.921e-06 | 0.010 |  |
| ebf9c7c66db43f6e2baab89cfdf12d41 | Chloroflexi | -37.556 | 1.494e-18 | 2.285e-15 | -10.669 | 1.705e-06 | 0.009 |  |
| af1bc90ea549e2ac70f4548e96fba5b4 | Chloroflexi | -22.600 | 1.157e-14 | 6.192e-12 | -9.413 | 2.830e-06 | 0.014 |  |
| 444c81850cce27f44ea33bb45738b6cb | Chloroflexi | -18.337 | 4.285e-13 | 1.638e-10 | -8.707 | 8.100e-06 | 0.030 |  |
| fa41a7b66551fa74a15eaed179ac235f | Chloroflexi | -26.342 | 7.923e-16 | 5.850e-13 | -7.918 | 9.423e-06 | 0.045 |  |
| 64e19b3d1da44d12d3fc023108260b1b | Chloroflexi | 14.558 | 2.125e-11 | 6.231e-09 | -7.833 | 4.650e-06 | 0.023 |  |
| 20db15662c1e2ef5df0cc0ba3b10f78d | Chloroflexi | -12.957 | 1.454e-10 | 3.890e-08 | -7.869 | 5.961e-06 | 0.028 |  |
| 642d2cb47bb7b74eb4ecaf2520460905 | Chloroflexia | -31.603 | 3.194e-17 | 3.799e-14 | -8.368 | 7.900e-06 | 0.039 |  |
| 510d788bd4475a3c40405cabf0fca233 | Holophagae | -20.659 | 5.501e-14 | 2.615e-11 | -8.219 | 6.451e-06 | 0.025 |  |
| 4bdb5af38bf69bad9f9fbbce2634e020 | Holophagae | -31.195 | 4.018e-17 | 4.200e-14 | -8.034 | 1.309e-05 | 0.043 |  |
| 87a6eefa6e996c377b6b5447504720ad | Thermoleophilia | -64.206 | 1.031e-22 | 8.291e-19 | -10.202 | 1.059e-06 | 0.005 |  |
| 6c6492ce2bad8705d41061ee5062a1eb | Chthoniobacterales | -17.711 | 7.762e-13 | 2.817e-10 | -8.386 | 3.465e-06 | 0.017 |  |
| 71addfb47679735a0fa84ec5aa1bab8f | Corynebacteriales | -8.559 | 9.237e-08 | 1.634e-05 | -6.957 | 2.924e-06 | 0.015 |  |
| 423b462d87749ef98eee20b8a58a710f | Thermomicrobiales | -63.779 | 1.162e-22 | 8.291e-19 | -12.126 | 2.349e-07 | 0.001 |  |
| aa548b25c86c045cab32a0b24b2473ab | Thermomicrobiales | -29.733 | 9.389e-17 | 9.137e-14 | -9.620 | 8.402e-06 | 0.026 |  |
| e8c311d8e25777f77b63be45d5191fd0 | Thermomicrobiales | -24.005 | 4.036e-15 | 2.469e-12 | -9.477 | 2.079e-05 | 0.015 |  |
| f7f63df19b681f488d289898f4fea4aa | Thermomicrobiales | -34.612 | 6.373e-18 | 9.096e-15 | -9.328 | 3.018e-06 | 0.015 |  |
| a90e585055a8254aae3e7805a0d8fca2 | Thermomicrobiales | -42.582 | 1.596e-19 | 3.417e-16 | -9.180 | 1.218e-05 | 0.024 |  |
| fbeeae7a656ec50dfd0a017d4e68b44e | Thermomicrobiales | -45.848 | 4.269e-20 | 1.142e-16 | -9.158 | 2.652e-06 | 0.013 |  |
| ea13fe9fdcd637af26569f5ca1a32946 | Thermomicrobiales | -31.199 | 4.010e-17 | 4.200e-14 | -8.743 | 1.302e-05 | 0.044 |  |
| 60d75dfe767bf6e0b3ace47dd76e4459 | Thermomicrobiales | -16.755 | 1.997e-12 | 6.785e-10 | -8.179 | 5.439e-06 | 0.027 |  |
| d2273237c6a9e43ce23119beba3bbf1f | Thermomicrobiales | -16.527 | 2.518e-12 | 8.294e-10 | -7.728 | 1.421e-05 | 0.044 |  |
| 21f1e8d9461147b629e97932631f3bf7 | Vicinamibacterales | -39.668 | 5.645e-19 | 9.296e-16 | -10.281 | 1.086e-06 | 0.005 |  |
| db6be6082965a96abbbc06b115af2d09 | Vicinamibacterales | -25.650 | 1.265e-15 | 8.735e-13 | -9.252 | 6.965e-06 | 0.030 |  |
| 914070a5921d7712507bbbfadba340ca | Vicinamibacterales | -27.215 | 4.468e-16 | 3.679e-13 | -9.108 | 7.635e-06 | 0.035 |  |
| 823b95422fffd15930389bd1200b42e4 | Vicinamibacterales | -20.490 | 6.338e-14 | 2.827e-11 | -8.659 | 7.493e-06 | 0.032 |  |
| c5a69722637cf33f1c6f3c2cec39ef7e | Vicinamibacterales | -18.550 | 3.519e-13 | 1.370e-10 | -8.189 | 7.384e-06 | 0.034 |  |
| 94f168bf1d4758457d5751b8e97a267d | Vicinamibacterales | -17.453 | 9.976e-13 | 3.600e-10 | -7.729 | 7.772e-06 | 0.039 |  |
| 6f125e1d73398f7407ee2d19c8082557 | Vicinamibacterales | -12.495 | 2.625e-10 | 6.691e-08 | -7.214 | 1.209e-05 | 0.046 |  |
| 60b681afbda0f74af05728635fff30af | Blastocatellaceae | -20.308 | 7.397e-14 | 3.232e-11 | -8.216 | 1.480e-05 | 0.039 |  |
| 0d3fdeeb2c85de64bf754f4f6c47803b | Micromonosporaceae | -60.063 | 3.407e-22 | 1.823e-18 | -9.282 | 1.917e-06 | 0.010 |  |
| 1d1fe3380e5ac53d8a062f10f5f4841f | Vicinamibacteraceae | -20.768 | 5.023e-14 | 2.501e-11 | -9.920 | 1.973e-05 | 0.019 |  |
| a3cab5660a856b609232b6e984a3def5 | *Bacillus* | -15.223 | 1.007e-11 | 3.124e-09 | -8.291 | 5.057e-06 | 0.025 |  |
| dc4fe3fd4e8747e9af0d924e24fb912a | *Brevibacillus* | -9.497 | 1.964e-08 | 4.043e-06 | -6.877 | 5.617e-06 | 0.028 |  |
| 66b16b98caa97191fc16dca99ad8fa13 | *Friedmanniella* | -40.952 | 3.199e-19 | 5.708e-16 | -8.886 | 7.389e-06 | 0.037 |  |
| a7ffc9fa81a7c3c515c0915a2081a037 | *Lihuaxuella* | -25.126 | 1.817e-15 | 1.144e-12 | -10.280 | 1.695e-06 | 0.008 |  |
| 6bec10ce7f7c55d25772e394e70747cc | *Microlunatus* | -97.906 | 5.299e-26 | 1.135e-21 | -14.139 | 1.797e-07 | 9.975e-04 |  |
| afae456fcd3b4cadc9a208a4f6f88a56 | *Polycladomyces* | -15.332 | 8.933e-12 | 2.813e-09 | -8.872 | 3.717e-06 | 0.019 |  |
| 4b8eea3e07f9e9ac389efb29bdbbe897 | *Rhodobaculum* | -48.905 | 1.348e-20 | 4.122e-17 | -11.115 | 2.628e-07 | 0.001 |  |
| 698e9071fa33454284e6b4ee8fec8eba | *Udaeobacter* | -15.100 | 1.153e-11 | 3.528e-09 | -8.047 | 5.693e-06 | 0.028 |  |
| 06b8eed98b034c36998df719045cf7b6 | *Udaeobacter* | -13.311 | 9.347e-11 | 2.599e-08 | -7.501 | 5.262e-06 | 0.026 |  |

Bacteria in sand for 6dS/m irrigation

| **ASV** | **Taxon** | ANCOMBC2 | | | ALDEx2 | | |  |
| --- | --- | --- | --- | --- | --- | --- | --- | --- |
|  |  | Statistic | p-value | p-adj | Statistic | p-value | p-adj |  |
| ecfe97812d3d226071f874708374d1dc | Gemmatimonadota | 5.315 | 4.723e-05 | 0.004 | 6.564 | 4.844e-06 | 0.030 |  |
| c6ea8fdb2562bae0809fba13a4d17809 | Comamonadaceae | 5.174 | 6.381e-05 | 0.006 | 7.177 | 1.451e-06 | 0.009 |  |
| 86fe369992720b5115fcb0e9426f9e8c | *Gemmatimonas* | 18.922 | 2.500e-13 | 1.071e-10 | 9.950 | 4.700e-06 | 0.024 |  |
| e05e972fcf21db0dd2a5bb6c5a74c0d1 | Chloroflexi | -68.830 | 2.963e-23 | 3.171e-19 | -11.855 | 5.321e-07 | 0.003 |  |
| ebf9c7c66db43f6e2baab89cfdf12d41 | Chloroflexi | -58.521 | 5.428e-22 | 2.324e-18 | -10.652 | 8.496e-06 | 0.014 |  |
| fa41a7b66551fa74a15eaed179ac235f | Chloroflexi | -35.620 | 3.827e-18 | 5.303e-15 | -8.395 | 6.392e-06 | 0.034 |  |
| af1bc90ea549e2ac70f4548e96fba5b4 | Chloroflexi | -33.466 | 1.158e-17 | 1.241e-14 | -9.934 | 3.064e-06 | 0.019 |  |
| d033a4fedc7e64d64bc61d34819d49e7 | Chloroflexi | -28.964 | 1.491e-16 | 1.277e-13 | -8.508 | 6.420e-06 | 0.035 |  |
| e8c311d8e25777f77b63be45d5191fd0 | Thermomicrobiales | -43.762 | 9.799e-20 | 2.331e-16 | -12.077 | 5.303e-07 | 0.003 |  |
| f7f63df19b681f488d289898f4fea4aa | Thermomicrobiales | -35.551 | 3.963e-18 | 5.303e-15 | -9.527 | 6.115e-06 | 0.020 |  |
| d2273237c6a9e43ce23119beba3bbf1f | Thermomicrobiales | -17.412 | 1.038e-12 | 3.644e-10 | -8.292 | 5.777e-06 | 0.026 |  |
| db6be6082965a96abbbc06b115af2d09 | Vicinamibacterales | -29.883 | 8.592e-17 | 7.665e-14 | -9.633 | 5.285e-06 | 0.031 |  |
| e2788f1cd3c0dac02a69515bbdb87bae | Vicinamibacterales | -28.558 | 1.912e-16 | 1.516e-13 | -9.097 | 6.380e-06 | 0.033 |  |
| 7dab1bb7affa4f2eb0e90ea8d588d9fb | Vicinamibacterales | -27.322 | 4.168e-16 | 2.975e-13 | -8.638 | 6.415e-06 | 0.035 |  |
| c5a69722637cf33f1c6f3c2cec39ef7e | Vicinamibacterales | -19.796 | 1.150e-13 | 5.237e-11 | -8.158 | 6.834e-06 | 0.040 |  |
| 78874c5be28095183acb705c6683a40c | Vicinamibacterales | -15.947 | 4.611e-12 | 1.496e-09 | -8.495 | 1.428e-05 | 0.048 |  |
| e029ced187206096f13dfc19115b1bfb | Microbacteriaceae | -14.155 | 3.389e-11 | 1.052e-08 | -10.664 | 7.920e-09 | 4.878e-05 |  |
| a3cab5660a856b609232b6e984a3def5 | *Bacillus* | -26.691 | 6.289e-16 | 4.343e-13 | -9.457 | 7.919e-06 | 0.033 |  |
| 66b16b98caa97191fc16dca99ad8fa13 | *Friedmanniella* | -48.018 | 1.869e-20 | 5.001e-17 | -9.280 | 2.339e-05 | 0.041 |  |
| 6bec10ce7f7c55d25772e394e70747cc | *Microlunatus* | -106.584 | 1.152e-26 | 2.466e-22 | -14.515 | 1.559e-08 | 9.553e-05 |  |
| d75f4944aa21ae15344deef09dd4cf50 | *Planifilum* | -10.841 | 2.543e-09 | 6.049e-07 | -8.326 | 1.425e-06 | 0.009 |  |
| 4b8eea3e07f9e9ac389efb29bdbbe897 | *Rhodobaculum* | -21.271 | 3.317e-14 | 1.614e-11 | -10.147 | 8.842e-07 | 0.005 |  |
| 06b8eed98b034c36998df719045cf7b6 | *Udaeobacter* | -38.297 | 1.056e-18 | 1.883e-15 | -11.099 | 8.129e-07 | 0.005 |  |
| 698e9071fa33454284e6b4ee8fec8eba | *Udaeobacter* | -31.524 | 3.338e-17 | 3.107e-14 | -10.118 | 3.501e-06 | 0.018 |  |
| 425dbf5cc6cbced5af9821fffc06e6ba | *Udaeobacter* | 24.396 | 3.042e-15 | 1.809e-12 | -8.338 | 1.039e-05 | 0.045 |  |
| 566a409979185fb9cdd650520863823c | *Udaeobacter* | -22.093 | 1.716e-14 | 8.963e-12 | -8.473 | 6.778e-06 | 0.035 |  |
| 4f183f8a070b55ba17f7c0d81b368c70 | *Udaeobacter* | -17.313 | 1.144e-12 | 3.951e-10 | -8.012 | 8.755e-06 | 0.036 |  |
| 91028e8d637c3af4b5b49be463515d31 | *Ureibacillus* | -12.204 | 3.845e-10 | 1.098e-07 | -7.639 | 1.125e-05 | 0.045 |  |

**Table S15.** Differential abundance of fungal mASVs before and after 9dS/m irrigation between treatments. The cells in red represent an increase in fungal abundance with higher salinity, while the cells in blue represent a decrease in bacterial abundance with higher salinity. Only mASVs showing an adjusted p-value < 0.05 (Bonferroni correction) for both the ANCOMBC2 and the ALDEx2 methods are presented. mASVs relate to those detailed in the ‘ITS_taxonomy.tsv’ and ‘ITS_reqseqs.fasta’ files.

Fungi in silt for 0dS/m irrigation

| **ASV** | **Taxon** | ANCOMBC2 | | | ALDEx2 | | |  |
| --- | --- | --- | --- | --- | --- | --- | --- | --- |
|  |  | Statistic | p-value | p-adj | Statistic | p-value | p-adj |  |
| 22691f5ff85679fe963241a0a1358956 | Fungi | 34.491 | 6.781e-18 | 2.688e-13 | 10.881 | 7.615e-06 | 0.015 |  |
| 5407141305220a31905dbf61448311db | Fungi | 26.701 | 6.245e-16 | 8.251e-12 | 9.746 | 1.713e-06 | 0.011 |  |
| 2826df1b1ab762fd943b491b2adfaa3a | Fungi | 16.642 | 2.240e-12 | 9.865e-09 | 8.937 | 7.974e-06 | 0.044 |  |
| 4ddf9bcb904d4ca55deb768087c6173e | Fungi | 20.567 | 5.940e-14 | 4.709e-10 | 8.827 | 3.996e-06 | 0.025 |  |
| 730db4dc5096737b83af4be7c087d93d | Fungi | 17.127 | 1.374e-12 | 6.810e-09 | 8.772 | 3.922e-06 | 0.025 |  |
| c340e7f5c25bc41eb6827db70e60e183 | Fungi | -18.376 | 4.132e-13 | 2.340e-09 | -9.520 | 5.633e-07 | 0.004 |  |
| b2d7454288f12252065e8f1b7e0dee62 | Fungi | -7.143 | 1.182e-06 | 0.002 | -7.489 | 7.168e-07 | 0.005 |  |
| 6f97c3a4861457ae243f8d0aea4308db | *Xenodidymella camporesii* | -21.855 | 2.073e-14 | 2.054e-10 | -11.201 | 5.041e-07 | 0.003 |  |

Fungi in silt for 3dS/m irrigation

| **ASV** | **Taxon** | ANCOMBC2 | | | ALDEx2 | | |  |
| --- | --- | --- | --- | --- | --- | --- | --- | --- |
|  |  | Statistic | p-value | p-adj | Statistic | p-value | p-adj |  |
| 2826df1b1ab762fd943b491b2adfaa3a | Fungi | 41.092 | 3.011e-19 | 1.194e-14 | 11.560 | 2.474e-07 | 0.002 |  |
| 15739d6f9a44fd40289e1bd96d3be9a1 | Fungi | 27.268 | 4.316e-16 | 3.333e-12 | 10.053 | 2.237e-06 | 0.014 |  |
| a357eaf9a5f3b1410fded755cc829259 | Fungi | 27.028 | 5.044e-16 | 3.333e-12 | 9.895 | 1.000e-05 | 0.028 |  |
| 730db4dc5096737b83af4be7c087d93d | Fungi | 23.919 | 4.299e-15 | 2.130e-11 | 9.831 | 1.808e-06 | 0.011 |  |
| 3cd2ccf3d3d3f03516140407a6131c2e | Fungi | 26.181 | 8.826e-16 | 4.998e-12 | 9.271 | 9.795e-06 | 0.021 |  |
| 5407141305220a31905dbf61448311db | Fungi | 17.022 | 1.526e-12 | 3.782e-09 | 9.171 | 4.948e-06 | 0.026 |  |
| e7b0c39a6bd9cc19f8b987a569d65046 | Fungi | 17.022 | 1.526e-12 | 3.782e-09 | 9.074 | 8.027e-06 | 0.040 |  |
| 508bbfa18f877e59d4089d6f0647b83f | Fungi | 38.784 | 8.428e-19 | 1.670e-14 | 8.988 | 3.643e-05 | 0.031 |  |
| 65f7eef6d2bd6b1aa178537671f5b311 | Fungi | 20.261 | 7.697e-14 | 2.179e-10 | 8.307 | 9.902e-06 | 0.045 |  |
| db1aaea1bcf78f4f91d512264d1c35da | Fungi | 28.091 | 2.558e-16 | 2.841e-12 | 8.235 | 5.713e-06 | 0.035 |  |
| 0e5537e9ef684d492eee9ecc4c0e5ccb | *Paecilomyces* | 23.265 | 6.975e-15 | 2.514e-11 | 10.930 | 5.606e-07 | 0.004 |  |
| 289ac2586a52904c6f73de8f7148897d | *Penicillium* | 22.435 | 1.314e-14 | 4.339e-11 | 10.243 | 9.707e-07 | 0.006 |  |
| f2860d907390be334e1bfd143bfc0c3b | Fungi | -14.818 | 1.580e-11 | 3.296e-08 | -9.134 | 2.086e-06 | 0.013 |  |
| f4a60b5e406305b230a8e2f74f5ecc5f | Fungi | -14.924 | 1.404e-11 | 3.091e-08 | -8.956 | 3.549e-06 | 0.022 |  |
| 0e952bdd65f0e231735cab357c0a9426 | Fungi | -13.186 | 1.091e-10 | 2.059e-07 | -8.280 | 3.916e-06 | 0.024 |  |
| c340e7f5c25bc41eb6827db70e60e183 | Fungi | -12.196 | 3.885e-10 | 6.999e-07 | -8.056 | 2.844e-06 | 0.018 |  |
| b2d7454288f12252065e8f1b7e0dee62 | Fungi | -6.631 | 3.175e-06 | 0.003 | -7.254 | 1.075e-06 | 0.007 |  |
| 797919d6753c33230959e9ca602e153f | Fungi | -9.750 | 1.317e-08 | 2.270e-05 | -6.999 | 7.877e-06 | 0.049 |  |
| b359c7c7fb7a9070713e311b34b8863a | Fungi | -5.700 | 2.095e-05 | 2.270e-05 | -6.545 | 3.994e-06 | 0.025 |  |
| dfa1fb44e44664ec439e05839411a400 | Fungi | -5.784 | 1.758e-05 | 0.010 | -6.390 | 5.728e-06 | 0.036 |  |
| 8c5b731eda26860d5808b0b35586c023 | Fungi | -5.197 | 6.078e-05 | 0.029 | -6.274 | 7.392e-06 | 0.046 |  |
| 0391afa7ea84ad686b40de169884e3e8 | *Acremonium* | -5.437 | 3.639e-05 | 0.019 | -6.272 | 6.640e-06 | 0.042 |  |
| 0bfdec6132fa1dc295df2c48ce33bbf7 | *Pseudeurotium* | -6.030 | 1.059e-05 | 0.007 | -6.844 | 2.150e-06 | 0.013 |  |
| 75bb0a46bdf2337c7454569347c9ba02 | *Chrysosporium lobatum* | -6.200 | 7.503e-06 | 0.006 | -6.953 | 1.823e-06 | 0.011 |  |
| 6f97c3a4861457ae243f8d0aea4308db | *Xenodidymella camporesii* | -23.719 | 4.979e-15 | 2.193e-11 | -11.306 | 1.126e-07 | 7.057e-04 |  |

Fungi in silt for 6dS/m irrigation

| **ASV** | **Taxon** | ANCOMBC2 | | | ALDEx2 | | |  |
| --- | --- | --- | --- | --- | --- | --- | --- | --- |
|  |  | Statistic | p-value | p-adj | Statistic | p-value | p-adj |  |
| 026d5cab8beb8b10a3458166df0fac98 | *Trichocladium* | -10.671 | 3.258e-09 | 1.291e-04 | -7.311 | 7.275e-06 | 0.044 |  |

Fungi in sand for 0dS/m irrigation

| **ASV** | **Taxon** | ANCOMBC2 | | | ALDEx2 | | |  |
| --- | --- | --- | --- | --- | --- | --- | --- | --- |
|  |  | Statistic | p-value | p-adj | Statistic | p-value | p-adj |  |
| a4e321c69e7d5c4a34b96741f982b56f | Fungi | -15.749 | 3.671e-11 | 1.455e-06 | -9.921 | 2.419e-06 | 0.017 |  |

Fungi in sand for 3dS/m irrigation

| **ASV** | **Taxon** | ANCOMBC2 | | | ALDEx2 | | |  |
| --- | --- | --- | --- | --- | --- | --- | --- | --- |
|  |  | Statistic | p-value | p-adj | Statistic | p-value | p-adj |  |
| c720f843fb4abbdf5b3cce81520fbdf6 | Tremellomycetes | 7.563 | 5.400e-07 | 0.004 | 6.858 | 2.937e-06 | 0.023 |  |
| a4e321c69e7d5c4a34b96741f982b56f | Fungi | -12.241 | 3.659e-10 | 1.451 | -7.672 | 8.435e-06 | 0.032 |  |

Fungi in sand for 6dS/m irrigation

| **ASV** | **Taxon** | ANCOMBC2 | | | ALDEx2 | | |  |
| --- | --- | --- | --- | --- | --- | --- | --- | --- |
|  |  | Statistic | p-value | p-adj | Statistic | p-value | p-adj |  |
| a4e321c69e7d5c4a34b96741f982b56f | Fungi | -20.850 | 4.691e-14 | 1.859e-09 | -9.812 | 6.238e-07 | 0.005 |  |

**Table S16.** Summary of significant bacterial mASV co-occurrences using the SCNIC method, with a p_adj_ < 0.05 and a r > 0.9 cut-off thresholds.

| Soil | Interactions | 0 dS/m | 3 dS/m | 6 dS/m |
| --- | --- | --- | --- | --- |
| Silt | Number of interactions pre-9 dS/m | 735 | 382 | 207 |
|  | **Number of same interactions pre-0 and pre-3 dS/m** | **16** | |  |
|  | **Percentage of same interactions pre-0 and pre-3 dS/m** | **1.442741208** | |  |
|  | **Number of same interactions pre-0 and pre-6 dS/m** |  |  | **3** |
|  | **Percentage of same interactions pre-0 and pre-6 dS/m** |  |  | **0.318979** |
|  | **Number of same interactions pre-3 and pre-6 dS/m** |  | **3** | |
|  | **Percentage of same interactions pre-3 and pre-6 dS/m** |  | **0.510638298** | |
|  | Number of interactions post-9 dS/m | 1954 | 2398 | 1941 |
|  | **Number of same interactions post-0 and post-3 dS/m** | **282** | |  |
|  | **Percentage of same interactions post-0 and post-3 dS/m** | **6.696746616** | |  |
|  | **Number of same interactions post-0 and post-6 dS/m** |  |  | **284** |
|  | **Percentage of same interactions post-0 and post-6 dS/m** |  |  | **7.56728** |
|  | **Number of same interactions post-3 and post-6 dS/m** |  | **222** | |
|  | **Percentage of same interactions post-3 and post-6 dS/m** |  | **5.250709555** | |
|  | **Number of same interactions pre- and post-9 dS/m** | **22** | **0** | **3** |
|  | **Percentage of same interactions pre- and post-9 dS/m** | **0.818148** | **0** | **0.139665** |
|  | Number of interactions post-9 dS/m compared to pre-9 dS/m | 2667 | 2780 | 2145 |
|  | Number of increase post-9 dS/m compared to pre-9 dS/m | 1941 | 2398 | 1939 |
|  | Number of decrease post-9 dS/m compared to pre-9 dS/m | 726 | 382 | 206 |
| Clay | Number of interactions pre-9 dS/m | 544 | 407 | 376 |
|  | **Number of same interactions pre-0 and pre-3 dS/m** | **44** | |  |
|  | **Percentage of same interactions pre-0 and pre-3 dS/m** | **4.736275565** | |  |
|  | **Number of same interactions pre-0 and pre-6 dS/m** |  |  | **14** |
|  | **Percentage of same interactions pre-0 and pre-6 dS/m** |  |  | **1.533406** |
|  | **Number of same interactions pre-3 and pre-6 dS/m** |  | **38** | |
|  | **Percentage of same interactions pre-3 and pre-6 dS/m** |  | **4.97382199** | |
|  | Number of interactions post-9 dS/m | 6695 | 196 | 8930 |
|  | **Number of same interactions post-0 and post-3 dS/m** | **27** | |  |
|  | **Percentage of same interactions post-0 and post-3 dS/m** | **0.392584515** | |  |
|  | **Number of same interactions post-0 and post-6 dS/m** |  |  | **4193** |
|  | **Percentage of same interactions post-0 and post-6 dS/m** |  |  | **30.99383** |
|  | **Number of same interactions post-3 and post-6 dS/m** |  | **30** | |
|  | **Percentage of same interactions post-3 and post-6 dS/m** |  | **0.329272308** | |
|  | **Number of same interactions pre- and post-9 dS/m** | **63** | **4** | **27** |
|  | **Percentage of same interactions pre- and post-9 dS/m** | **0.870286** | **0.66335** | **0.290135** |
|  | Number of interactions post-9 dS/m compared to pre-9 dS/m | 7176 | 599 | 9279 |
|  | Number of increase post-9 dS/m compared to pre-9 dS/m | 6676 | 194 | 8922 |
|  | Number of decrease post-9 dS/m compared to pre-9 dS/m | 500 | 405 | 357 |
| Sand | Number of interactions pre-9 dS/m | 325 | 327 | 326 |
|  | **Number of same interactions pre-0 and pre-3 dS/m** | **38** | |  |
|  | **Percentage of same interactions pre-0 and pre-3 dS/m** | **6.003159558** | |  |
|  | **Number of same interactions pre-0 and pre-6 dS/m** |  |  | **21** |
|  | **Percentage of same interactions pre-0 and pre-6 dS/m** |  |  | **3.278689** |
|  | **Number of same interactions pre-3 and pre-6 dS/m** |  | **15** | |
|  | **Percentage of same interactions pre-3 and pre-6 dS/m** |  | **2.323780015** | |
|  | Number of interactions post-9 dS/m | 8359 | 206 | 2623 |
|  | **Number of same interactions post-0 and post-3 dS/m** | **33** | |  |
|  | **Percentage of same interactions post-0 and post-3 dS/m** | **0.386032637** | |  |
|  | **Number of same interactions post-0 and post-6 dS/m** |  |  | **1219** |
|  | **Percentage of same interactions post-0 and post-6 dS/m** |  |  | **11.75223** |
|  | **Number of same interactions post-3 and post-6 dS/m** |  | **15** | |
|  | **Percentage of same interactions post-3 and post-6 dS/m** |  | **0.531632111** | |
|  | **Number of same interactions pre- and post-9 dS/m** | **34** | **6** | **48** |
|  | **Percentage of same interactions pre- and post-9 dS/m** | **0.391525** | **1.125704** | **1.62767** |
|  | Number of interactions post-9 dS/m compared to pre-9 dS/m | 8650 | 527 | 2901 |
|  | Number of increase post-9 dS/m compared to pre-9 dS/m | 8351 | 202 | 2596 |
|  | Number of decrease post-9 dS/m compared to pre-9 dS/m | 299 | 325 | 305 |

**Table S17.** Summary of significant fungal mASV co-occurrences using the SCNIC method, with a p_adj_ < 0.05 and a r > 0.9 cut-off thresholds.

| Soil | Interactions | 0 dS/m | 3 dS/m | 6 dS/m |
| --- | --- | --- | --- | --- |
| Silt | Number of interactions pre-9 dS/m | 129 | 47 | 29 |
|  | **Number of same interactions pre-0 and pre-3 dS/m** | **5** | |  |
|  | **Percentage of same interactions pre-0 and pre-3 dS/m** | **2.88184438** | |  |
|  | **Number of same interactions pre-0 and pre-6 dS/m** |  |  | **5** |
|  | **Percentage of same interactions pre-0 and pre-6 dS/m** |  |  | **3.215434** |
|  | **Number of same interactions pre-3 and pre-6 dS/m** |  | **4** | |
|  | **Percentage of same interactions pre-3 and pre-6 dS/m** |  | **5.405405405** | |
|  | Number of interactions post-9 dS/m | 547 | 383 | 820 |
|  | **Number of same interactions post-0 and post-3 dS/m** | **5** | |  |
|  | **Percentage of same interactions post-0 and post-3 dS/m** | **0.539083558** | |  |
|  | **Number of same interactions post-0 and post-6 dS/m** |  |  | **75** |
|  | **Percentage of same interactions post-0 and post-6 dS/m** |  |  | **5.641219** |
|  | **Number of same interactions post-3 and post-6 dS/m** |  | **13** | |
|  | **Percentage of same interactions post-3 and post-6 dS/m** |  | **1.086502298** | |
|  | **Number of same interactions pre- and post-9 dS/m** | **3** | **2** | **1** |
|  | **Percentage of same interactions pre- and post-9 dS/m** | **0.443787** | **0.465116** | **0.117786** |
|  | Number of interactions post-9 dS/m compared to pre-9 dS/m | 673 | 429 | 848 |
|  | Number of increase post-9 dS/m compared to pre-9 dS/m | 547 | 382 | 82 |
|  | Number of decrease post-9 dS/m compared to pre-9 dS/m | 126 | 47 | 28 |
| Clay | Number of interactions pre-9 dS/m | 68 | 138 | 161 |
|  | **Number of same interactions pre-0 and pre-3 dS/m** | **12** | |  |
|  | **Percentage of same interactions pre-0 and pre-3 dS/m** | **6** | |  |
|  | **Number of same interactions pre-0 and pre-6 dS/m** |  |  | **22** |
|  | **Percentage of same interactions pre-0 and pre-6 dS/m** |  |  | **10.09174** |
|  | **Number of same interactions pre-3 and pre-6 dS/m** |  | **49** | |
|  | **Percentage of same interactions pre-3 and pre-6 dS/m** |  | **17.85063752** | |
|  | Number of interactions post-9 dS/m | 1699 | 38 | 1617 |
|  | **Number of same interactions post-0 and post-3 dS/m** | **6** | |  |
|  | **Percentage of same interactions post-0 and post-3 dS/m** | **0.346020761** | |  |
|  | **Number of same interactions post-0 and post-6 dS/m** |  |  | **452** |
|  | **Percentage of same interactions post-0 and post-6 dS/m** |  |  | **14.62783** |
|  | **Number of same interactions post-3 and post-6 dS/m** |  | **4** | |
|  | **Percentage of same interactions post-3 and post-6 dS/m** |  | **0.241984271** | |
|  | **Number of same interactions pre- and post-9 dS/m** | **16** | **2** | **18** |
|  | **Percentage of same interactions pre- and post-9 dS/m** | **0.90549** | **1.136364** | **1.012373** |
|  | Number of interactions post-9 dS/m compared to pre-9 dS/m | 1751 | 174 | 1760 |
|  | Number of increase post-9 dS/m compared to pre-9 dS/m | 1694 | 38 | 1614 |
|  | Number of decrease post-9 dS/m compared to pre-9 dS/m | 57 | 136 | 146 |
| Sand | Number of interactions pre-9 dS/m | 36 | 39 | 46 |
|  | **Number of same interactions pre-0 and pre-3 dS/m** | **1** | |  |
|  | **Percentage of same interactions pre-0 and pre-3 dS/m** | **1.342281879** | |  |
|  | **Number of same interactions pre-0 and pre-6 dS/m** |  |  | **0** |
|  | **Percentage of same interactions pre-0 and pre-6 dS/m** |  |  | **0** |
|  | **Number of same interactions pre-3 and pre-6 dS/m** |  | **0** | |
|  | **Percentage of same interactions pre-3 and pre-6 dS/m** |  | **0** | |
|  | Number of interactions post-9 dS/m | 1155 | 38 | 285 |
|  | **Number of same interactions post-0 and post-3 dS/m** | **2** | |  |
|  | **Percentage of same interactions post-0 and post-3 dS/m** | **0.167785235** | |  |
|  | **Number of same interactions post-0 and post-6 dS/m** |  |  | **43** |
|  | **Percentage of same interactions post-0 and post-6 dS/m** |  |  | **3.031371** |
|  | **Number of same interactions post-3 and post-6 dS/m** |  | **4** | |
|  | **Percentage of same interactions post-3 and post-6 dS/m** |  | **1.246105919** | |
|  | **Number of same interactions pre- and post-9 dS/m** | **0** | **0** | **2** |
|  | **Percentage of same interactions pre- and post-9 dS/m** | **0** | **0** | **0.60423** |
|  | Number of interactions post-9 dS/m compared to pre-9 dS/m | 1191 | 77 | 329 |
|  | Number of increase post-9 dS/m compared to pre-9 dS/m | 1155 | 38 | 284 |
|  | Number of decrease post-9 dS/m compared to pre-9 dS/m | 36 | 39 | 45 |

Analyses underlying the statistical outputs for tables S18 and S19 are in ‘Script4_yield_analyses.R’

**Table S18.** Marketable potato yield parameters. Marketable potatoes are those that are greater than 45 mm in diameter

| **Mesocosm number** | **Irrigation treatment** | **Soil** | **Number of tubers** | **Mean tuber size (mm)** | **Mean tuber fresh weight (g)** | **Total tuber fresh weight (g)** | **Total tuber dry weight (g)** |
| --- | --- | --- | --- | --- | --- | --- | --- |
| 1 | 3dS/m | silt | 7 | 51.14 | 42.25 | 295.72 | 102.09 |
| 5 | 6dS/m | silt | 6 | 52.67 | 35.26 | 211.53 | 59.30 |
| 6 | 3dS/m | silt | 7 | 49.43 | 36.98 | 258.87 | 100.64 |
| 7 | 0dS/m | silt | 8 | 49.88 | 36.45 | 291.6 | 143.64 |
| 9 | 3dS/m | silt | 6 | 49.67 | 36.69 | 220.11 | 121.00 |
| 12 | 6dS/m | silt | 5 | 53.00 | 42.35 | 211.74 | 82.88 |
| 13 | 3dS/m | silt | 8 | 53.50 | 46.34 | 370.68 | 153.46 |
| 16 | 6dS/m | silt | 3 | 56.00 | 47.38 | 142.13 | 60.40 |
| 17 | 0dS/m | silt | 5 | 59.60 | 58.84 | 294.2 | 97.56 |
| 19 | 6dS/m | silt | 4 | 45.75 | 29.49 | 117.94 | 47.54 |
| 20 | 0dS/m | silt | 8 | 54.38 | 45.33 | 362.65 | 148.48 |
| 24 | 6dS/m | silt | 5 | 59.00 | 63.73 | 318.64 | 154.77 |
| 26 | 3dS/m | silt | 3 | 50.00 | 40.52 | 121.55 | 107.99 |
| 28 | 0dS/m | silt | 4 | 53.75 | 45.91 | 183.62 | 120.70 |
| 30 | 0dS/m | silt | 6 | 59.67 | 66.91 | 401.46 | 238.21 |
| 31 | 3dS/m | clay | 5 | 53.00 | 58.52 | 292.58 | 111.74 |
| 35 | 6dS/m | clay | 6 | 54.17 | 51.86 | 311.14 | 100.07 |
| 36 | 3dS/m | clay | 4 | 52.50 | 48.18 | 192.73 | 127.02 |
| 37 | 0dS/m | clay | 6 | 53.50 | 43.72 | 262.34 | 123.71 |
| 39 | 3dS/m | clay | 5 | 56.00 | 58.19 | 290.97 | 163.59 |
| 42 | 6dS/m | clay | 6 | 57.50 | 62.30 | 373.82 | 146.13 |
| 43 | 3dS/m | clay | 10 | 57.00 | 37.48 | 374.82 | 118.84 |
| 46 | 6dS/m | clay | 7 | 52.14 | 49.07 | 343.46 | 181.36 |
| 47 | 0dS/m | clay | 6 | 54.17 | 54.14 | 324.86 | 150.88 |
| 49 | 6dS/m | clay | 5 | 71.60 | 89.31 | 446.57 | 118.19 |
| 50 | 0dS/m | clay | 7 | 49.00 | 40.05 | 280.35 | 121.28 |
| 54 | 6dS/m | clay | 4 | 61.25 | 77.69 | 310.76 | 126.47 |
| 56 | 3dS/m | clay | 5 | 49.60 | 43.47 | 217.34 | 140.52 |
| 58 | 0dS/m | clay | 6 | 52.17 | 49.58 | 297.5 | 112.84 |
| 60 | 0dS/m | clay | 5 | 57.20 | 59.94 | 299.69 | 125.72 |
| 61 | 3dS/m | sand | 11 | 54.64 | 38.52 | 423.72 | 105.50 |
| 65 | 6dS/m | sand | 6 | 60.50 | 61.17 | 367.02 | 66.82 |
| 66 | 3dS/m | sand | 9 | 49.56 | 34.70 | 312.27 | 88.90 |
| 67 | 0dS/m | sand | 10 | 51.10 | 38.71 | 387.14 | 133.89 |
| 69 | 3dS/m | sand | 7 | 62.14 | 65.49 | 458.45 | 127.24 |
| 72 | 6dS/m | sand | 5 | 55.00 | 50.37 | 251.83 | 106.18 |
| 73 | 3dS/m | sand | 6 | 66.67 | 61.42 | 368.5 | 158.48 |
| 76 | 6dS/m | sand | 5 | 55.00 | 55.25 | 276.27 | 87.77 |
| 77 | 0dS/m | sand | 11 | 54.36 | 40.30 | 443.33 | 164.57 |
| 79 | 6dS/m | sand | 8 | 51.88 | 50.55 | 404.37 | 137.86 |
| 80 | 0dS/m | sand | 7 | 61.43 | 68.87 | 482.06 | 178.02 |
| 84 | 6dS/m | sand | 9 | 51.11 | 42.99 | 386.95 | 169.36 |
| 86 | 3dS/m | sand | 10 | 53.30 | 42.53 | 425.3 | 124.98 |
| 88 | 0dS/m | sand | 9 | 59.44 | 49.99 | 449.95 | 149.12 |
| 90 | 0dS/m | sand | 11 | 56.50 | 53.31 | 586.4 | 212.17 |

**Table S19.** Spinach emergence and yield data. Five seeds were planted in each mesocosm.

| Mesocosm number | Irrigation treatment | Soil type | Number emerged first planting | Total fresh weight Aug harvest  (g) | Mean fresh weight of those emerged Aug harvest  (g) | Total dry weight Aug harvest  (g) | Mean dry weight of those emerged Aug harvest (g) | Number emerged second planting | Total fresh weight Sept harvest (g) | Mean fresh weight of those emerged Sept harvest (g) | Total dry weight Sept harvest  (g) | Mean dry weight of those emerged Sept harvest (g) |
| --- | --- | --- | --- | --- | --- | --- | --- | --- | --- | --- | --- | --- |
| 2 | 3dS/m | silt | 2 | 164.72 | 82.36 | 10.70 | 5.35 | 3 | 41.14 | 13.71 | 2.97 | 0.99 |
| 3 | 0dS/m | silt | 4 | 274.90 | 68.73 | 39.85 | 9.96 | 4 | 94.05 | 23.51 | 7.36 | 1.84 |
| 4 | 6dS/m | silt | 1 | 69.60 | 69.60 | 4.27 | 4.27 | 0 | 0 | 0 | 0 | 0 |
| 8 | 3dS/m | silt | 2 | 145.06 | 72.53 | 10.69 | 5.35 | 2 | 40.72 | 20.36 | 2.89 | 1.45 |
| 10 | 6dS/m | silt | 1 | 54.45 | 54.45 | 4.02 | 4.02 | 0 | 0 | 0 | 0 | 0 |
| 11 | 0dS/m | silt | 4 | 266.06 | 66.52 | 16.22 | 4.06 | 4 | 91.81 | 22.95 | 6.23 | 1.56 |
| 14 | 0dS/m | silt | 5 | 317.83 | 63.57 | 14.78 | 2.96 | 4 | 98.00 | 24.50 | 7.18 | 1.80 |
| 15 | 0dS/m | silt | 2 | 158.87 | 79.44 | 0.71 | 0.36 | 4 | 141.23 | 35.31 | 10.21 | 2.55 |
| 18 | 3dS/m | silt | 2 | 197.58 | 98.79 | 13.64 | 6.82 | 4 | 41.47 | 10.37 | 3.05 | 0.76 |
| 21 | 3dS/m | silt | 3 | 214.75 | 71.58 | 13.39 | 4.46 | 1 | 11.86 | 11.86 | 0.76 | 0.76 |
| 22 | 6dS/m | silt | 2 | 58.76 | 29.38 | 4.13 | 2.07 | 0 | 0 | 0 | 0 | 0 |
| 23 | 6dS/m | silt | 0 | 0 | 0 | 0 | 0 | 0 | 0 | 0 | 0 | 0 |
| 25 | 3dS/m | silt | 2 | 185.11 | 92.56 | 8.53 | 4.27 | 4 | 63.69 | 15.92 | 4.74 | 1.19 |
| 27 | 6dS/m | silt | 2 | 60.64 | 30.32 | 3.45 | 1.73 | 0 | 0 | 0 | 0 | 0 |
| 29 | 0dS/m | silt | 3 | 183.74 | 61.25 | 11.55 | 3.85 | 4 | 43.44 | 10.86 | 3.37 | 0.84 |
| 32 | 3dS/m | clay | 5 | 94.30 | 18.86 | 5.26 | 1.05 | 4 | 37.48 | 9.37 | 2.88 | 0.72 |
| 33 | 0dS/m | clay | 5 | 116.79 | 23.36 | 7.04 | 1.41 | 5 | 60.89 | 12.18 | 4.91 | 0.98 |
| 34 | 6dS/m | clay | 2 | 46.77 | 23.39 | 3.45 | 1.73 | 2 | 18.91 | 9.46 | 1.50 | 0.75 |
| 38 | 3dS/m | clay | 4 | 133.25 | 33.31 | 9.03 | 2.26 | 4 | 31.15 | 7.79 | 2.23 | 0.56 |
| 40 | 6dS/m | clay | 2 | 34.88 | 17.44 | 1.59 | 0.80 | 2 | 14.36 | 7.18 | 1.25 | 0.63 |
| 41 | 0dS/m | clay | 5 | 150.16 | 30.03 | 10.84 | 2.17 | 5 | 53.84 | 10.77 | 4.01 | 0.80 |
| 44 | 0dS/m | clay | 2 | 52.67 | 26.34 | 3.12 | 1.56 | 5 | 68.86 | 13.77 | 5.37 | 1.07 |
| 45 | 0dS/m | clay | 4 | 94.15 | 23.54 | 5.71 | 1.43 | 5 | 48.30 | 9.66 | 3.75 | 0.75 |
| 48 | 3dS/m | clay | 3 | 93.43 | 31.14 | 6.41 | 2.14 | 3 | 42.78 | 14.26 | 3.52 | 1.17 |
| 51 | 3dS/m | clay | 4 | 123.03 | 30.76 | 6.56 | 1.64 | 4 | 33.13 | 8.28 | 2.58 | 0.65 |
| 52 | 6dS/m | clay | 2 | 49.47 | 24.74 | 3.25 | 1.63 | 2 | 8.21 | 4.11 | 1.22 | 0.61 |
| 53 | 6dS/m | clay | 1 | 20.50 | 20.50 | 2.19 | 2.19 | 2 | 9.82 | 4.91 | 0.79 | 0.40 |
| 55 | 3dS/m | clay | 5 | 114.41 | 22.88 | 6.35 | 1.27 | 3 | 40.03 | 13.34 | 2.89 | 0.96 |
| 57 | 6dS/m | clay | 3 | 105.53 | 35.18 | 6.21 | 2.07 | 2 | 3.78 | 1.89 | 0.46 | 0.23 |
| 59 | 0dS/m | clay | 5 | 90.81 | 18.16 | 9.86 | 1.97 | 4 | 42.68 | 10.67 | 3.62 | 0.91 |
| 62 | 3dS/m | sand | 3 | 65.35 | 21.78 | 4.59 | 1.53 | 3 | 20.75 | 6.92 | 1.61 | 0.54 |
| 63 | 0dS/m | sand | 3 | 95.52 | 31.84 | 5.96 | 1.99 | 5 | 55.45 | 11.09 | 4.85 | 0.97 |
| 64 | 6dS/m | sand | 2 | 45.71 | 22.86 | 1.90 | 0.95 | 1 | 11.06 | 11.06 | 1.22 | 1.22 |
| 68 | 3dS/m | sand | 2 | 63.04 | 31.52 | 4.06 | 2.03 | 2 | 17.29 | 8.65 | 1.58 | 0.79 |
| 70 | 6dS/m | sand | 3 | 34.70 | 11.57 | 2.14 | 0.71 | 2 | 11.18 | 5.59 | 0.99 | 0.50 |
| 71 | 0dS/m | sand | 4 | 122.81 | 30.70 | 8.07 | 2.02 | 4 | 43.86 | 10.97 | 3.70 | 0.93 |
| 74 | 0dS/m | sand | 2 | 18.57 | 9.29 | 1.42 | 0.71 | 4 | 42.54 | 10.64 | 3.65 | 0.91 |
| 75 | 0dS/m | sand | 3 | 135.05 | 45.02 | 6.95 | 2.32 | 2 | 28.27 | 14.14 | 2.26 | 1.13 |
| 78 | 3dS/m | sand | 4 | 13.17 | 3.29 | 2.54 | 0.64 | 3 | 2.22 | 0.74 | 0.50 | 0.17 |
| 81 | 3dS/m | sand | 3 | 0.87 | 0.29 | 0.29 | 0.10 | 4 | 6.96 | 1.74 | 0.72 | 0.18 |
| 82 | 6dS/m | sand | 1 | 1.30 | 1.30 | 0.23 | 0.23 | 2 | 2.90 | 1.45 | 0.30 | 0.15 |
| 83 | 6dS/m | sand | 2 | 11.76 | 5.88 | 1.12 | 0.56 | 1 | 0.12 | 0.12 | 0.62 | 0.62 |
| 85 | 3dS/m | sand | 3 | 7.24 | 2.41 | 0.53 | 0.18 | 3 | 16.15 | 5.38 | 1.43 | 0.48 |
| 87 | 6dS/m | sand | 2 | 37.67 | 18.84 | 2.80 | 1.40 | 2 | 0.04 | 0.02 | 0.09 | 0.05 |
| 89 | 0dS/m | sand | 2 | 5.52 | 2.76 | 0.44 | 0.22 | 4 | 28.18 | 7.05 | 2.41 | 0.60 |
